# Framing the futures of animal-free dairy: Using focus groups to explore early-adopter perceptions of the precision fermentation process

**DOI:** 10.3389/fnut.2022.997632

**Published:** 2022-10-03

**Authors:** Garrett M. Broad, Oscar Zollman Thomas, Courtney Dillard, Daniel Bowman, Brice Le Roy

**Affiliations:** ^1^Department of Communication Studies, Rowan University, Glassboro, NJ, United States; ^2^Formo, Berlin, Germany; ^3^Mercy For Animals, Los Angeles, CA, United States; ^4^School of English, The University of Sheffield, Sheffield, United Kingdom

**Keywords:** animal-free dairy, precision fermentation, consumer perceptions, alternative proteins, framing, food technology

## Abstract

This paper reports on the findings from a series of virtual focus groups that explored consumer perceptions of animal-free dairy (AFD), an emerging type of animal product alternative produced using the tools of synthetic biology and precision fermentation. Focus group participants came from an international sample of potential “early adopters.” To stimulate conversation, participants were presented with a series of visual “moodboards” that framed key arguments both in favor of and in opposition to AFD. Three primary thematic clusters emerged from the discussion. The first focused on issues of “process, safety, and regulation,” centered on the general reaction of participants to the concept of AFD, their primary concerns, key questions, and the assurances they would need in order to support its advancement. The second focused on issues of “consumer preferences and priorities,” highlighted by the often complicated, and sometimes outright contradictory, stated consumer interests of the participants. The third focused on issues of “food technology and the future,” wherein participants expressed broader views on the role of food technology in society, generally speaking, and the potential futures of AFD, specifically. The general consensus among participants was a cautious openness to the idea of AFD. Outright opposition to the concept was rare, but so too was unabashed enthusiasm. Instead, respondents had a number of questions about the nature of the technological process, its overall safety and regulatory standards, its potential contributions to individual health and climate change mitigation, as well as its organoleptic qualities and price to consumers. Among the positive frames, claims about animal welfare were deemed the most pertinent and convincing. Among the negative frames, concerns about messing with nature and creating potential health risks to individuals were seen as the strongest arguments against AFD. The findings suggest that the key to AFD's future as a viable market option will depend in large part on the extent to which it can clearly demonstrate that it is preferable to conventional dairy or its plant-based competitors, particularly in the arena of taste, but also across considerations of health and safety, nutrition, environmental effects, and animal well-being.

## Introduction

In June of 2022, a joint press release from the animal-free dairy startup Perfect Day and the confectionery multinational Mars, Incorporated announced the launch of a “new sustainability inspired chocolate experience, CO2COA™.” The product was touted as the Mars company's “first ever earth-friendly and animal-free chocolate innovation,” with a name that gave a nod to the product's Rainforest Alliance-certified cocoa and the reduction in greenhouse gas emissions (CO2) that came from product sourcing. The press release described Perfect Day's flagship product as the world's first precision-fermented protein, developed by utilizing “microflora to create proprietary animal-free milk protein” (1). On that very same day, the Non-GMO Project – a non-profit organization that advocates against GM foods and promotes its independent non-GMO label standard – held a webinar entitled, “How do you milk a microbe? How synbio is disrupting the dairy industry.” There, a series of panelists raised a host of concerns about the emerging technology and industry of animal-free dairy, critiquing it for a lack of transparency, raising questions about unknown health and environmental impacts, and insisting that it represented the wrong approach for the future of dairy and the food system. As promotional materials for the event put it: “These new GMOs are largely unregulated and unlabeled, and they're flying under the radar of the natural products industry” (2).

These dueling announcements made it clear that a “frame contest” over animal-free dairy (AFD) – an emerging type of animal product alternative produced using the tools of synthetic biology and precision fermentation – had begun (3). The aim of this paper is to provide early-stage insight into that question, as it reports on the findings from a series of virtual focus groups held on the topic of AFD that explored consumer perceptions and reactions to positive and negative frames about the technology and its implications. The research was the product of a multi-sectoral partnership that included an academic researcher, a researcher from an animal protection non-profit, and a researcher from an AFD company, as well as several other collaborators in supporting roles. Focus group participants came from an international sample of potential “early adopters,” with representation from the United States, United Kingdom, Germany, and Singapore.

The primary goal of the research was to understand how potential consumers make sense of this new way to make dairy, to explore their general level of interest and concern regarding the technology, to see what types of positive or negative arguments about AFD resonate as convincing or pertinent, and to interrogate what questions they have about the process and its implications. The project's conceptual and methodological foundations were grounded in framing theory, a wide-ranging approach that is based in the recognition that a single issue can be viewed from a variety of perspectives, as well as construed as having implications for different sets of values (4). The paper proceeds from here by outlining the broader landscape of animal product alternatives and situates AFD within existing research and practice on the topic. It outlines the conceptual and methodological approach of the research process, then details the key thematic takeaways of the focus groups. It concludes by offering reflections on the work's implications in the areas of research, advocacy, and private sector consumer engagement.

### Animal product alternatives and animal-free dairy

The category of animal product analogs and alternatives has experienced notable growth over the past several decades. While there is a long and storied history of food products that are intended to substitute for conventional meat, dairy, or eggs, in recent years there has been significant financial and human capital invested to promote innovation and market expansion in this arena (5). A variety of advocates believe that new technological and market-based developments in alternative proteins – that which can draw from a mix of plant-based, fermentation-based, and cell-cultivation approaches to create products that closely mimic or are compositionally identical to their animal counterparts – could have a transformative impact on local and global food systems (6). The impetus for this focus involves several key considerations, including concerns about the environmental and climate-related impacts of industrialized animal food production, the nutritional and public health costs of animal-derived products, and the ethical problems with respect to the treatment of animals in large-scale production processes, among other considerations (7). Across each of these topics, of course, there is widespread contestation regarding the merits and drawbacks of industrial animal food production, as well as of alternative or small-scale animal production, and the promotion of non-animal analogs.

The global market for meat substitutes – which primarily consists of a mix of minimally processed soy products like tofu and tempeh, processed vegetable protein and wheat analogs, and fermented mycoprotein products – was valued at approximately $5.5 billion in 2020 and is projected to reach $11.2 billion by 2030 (8). The global dairy alternatives market – which uses plant-based ingredients such as soy, almonds, oats, and rice to create analogs for milk, yogurt, ice cream, cheese, and other products – was estimated at $22.6 billion in 2020 and is projected to reach $40.6 billion by 2026 (9). In the United States, plant-based milk alternatives make up an estimated 10–15% of the entire milk category. An estimated 67% of adults have tried a non-dairy milk, approximately one in three adults drink alternative milks at least once per week, and the product is stocked in approximately 12% of US households, which is third in the category behind only whole and 2% milk (10, 11).

At the same time as these plant-based analogs have increased in prominence, a parallel science and industry called *cellular agriculture* has emerged as a new player in the animal product alternative landscape. The primary concept behind cellular agriculture is to use the tools of synthetic biology and tissue engineering to create products that are molecularly identical to conventionally produced animal foods without the need for raising and slaughtering animals. The field can be broadly divided into cellular products (made from live or formerly live cells) or acellular products (not containing cells). Cell-cultured (also called cultivated) meat production falls into the former category, with the process working through acquiring stem cells from an animal, encouraging those cells to grow by feeding them a cell-culture nutrient medium in a bioreactor, and then harvesting and assembling them into final meat analog products.

Acellular products, such as milk ingredients, can be created via similar techniques by culturing milk-producing mammary cells, which in turn produce milk. However, the foremost approach is through precision fermentation, wherein microorganisms (such as yeast) can be genetically programmed to express specific proteins, then mixed with nutrients and sugars in a bioreactor until those proteins are produced. From there, proteins are mixed with minerals, sugar, water, and plant-based (or, potentially, fermentation-based) fats to create “animal-free dairy” (AFD) products such as milk and ice cream (12–14). The production of this AFD is actually an extension of well-established processes of precision fermentation that have been used to create products such as synthetic insulin for diabetic treatment and genetically engineered rennet for mainstream cheese production (15). Indeed, in recent years these same acellular techniques can and have been used to produce non-animal food products such as vanillin (a primary component of vanilla flavor) as well as non-food products used in cosmetics, materials, and other related industries.

Both plant-based and cell-cultured products have been explored from a variety of scholarly angles, with researchers considering their key promises, potentials, and drawbacks from a host of critical, empirical, and philosophical perspectives. A significant body of research has focused on consumer perceptions and preferences related to these products (16). Much of this inquiry confirms what has long been understood about how and why consumers choose to eat or avoid particular foods. That is to say, consumer perceptions of food safety, taste, price, and nutrition are consistently found to be, on average, the most important factors that consumers note as determinants of their food choices. A host of other intersecting factors, including but not limited to convenience, hunger and physiological needs, emotional status, social dynamics and tradition, and the appearance of food also play a significant role in food decision-making. In addition, value-oriented considerations such as environmental impacts, labor practices, food origin, and perceived naturalness can be a significant factor for some consumers (17–19).

With that said, a persistent “attitude-behavior gap,” in which there is a disconnect between one's stated value-oriented preferences for sustainable foods, on one hand, and actual eating habits, on the other, has long been observed. This gap could be explained as the result of socially desirable misstatements from consumers, or alternately as the product of food environments that present too many obstacles to desired food purchasing (20). When it comes to the topic of innovative food technologies and products, their perceived benefits, risks, and naturalness are central to consumer perception. Attitudes toward innovative foods are influenced by surrounding social, economic, and political environments, such that trust in relevant food system institutions and a belief in the benefits of a new food product will be predictive of consumer openness and interest (21, 22).

With respect to plant-based animal product alternatives, specifically, research shows that purchasing intent is driven first and foremost by taste, followed by a sense that products appear familiar and match traditional meat or dairy counterparts (15). Health and nutrition, as well as altruistic benefits such as improved animal welfare and environmental benefits, matter more for particular consumer segments that have specific interest in these issues, as compared to less value-driven eaters (23). While those most likely to eat plant-based meat products are young, college educated, wealthier than average, and vegetarian, a nationally representative consumer survey from the International Food Information Council (24) found that nearly 50% of the sample of US adults had tried a meatless alternative, with the most cited motivation being a general interest in trying new foods. Other leading factors included curiosity; taste; an effort to eat less meat; as well as a belief that the alternatives are better for the environment, animals, or their personal health. Notably, concern about not liking the taste was cited as the main reason why respondents had not tried a plant-based meat alternative. Research into motivations for trying plant-based dairy alternatives has found similar results, confirming general curiosity about taste and perceived health benefits as primary drivers, alongside the influence of close friends and family (25). For some consumers, the perception that plant-based dairy is better than conventional in terms of greenhouse gas emissions or animal welfare also plays a role (26).

Despite the fact that cell-cultured meats are not yet a widely available consumer product, there have been a number of investigations into consumer perceptions of the topic. Indeed, several systematic reviews have already been published that summarize key findings related to consumer interest and concerns. Bryant and Barnett (27) found evidence to suggest that most consumers would try cell-cultured meat, but not necessarily use it as a replacement for conventional meat on a regular basis. Their review also suggested that younger people, men, educated consumers, people who eat meat (as opposed to vegetarians), and those familiar with the concept were more open to the product. Major barriers to acceptance included perceived unnaturalness, safety concerns, nutrition concerns, and questions about taste and price. They noted that attitudes toward cultured meat could be improved with information about its benefits, and highlighted the importance of targeted framing in this process. A review from Pakseresht et al. (28) found that the acceptance level of cultured meat is relatively low, that there are significant cross-cultural differences in consumer response, and that environmental and animal welfare advantages do not appear strong enough motivators to convince heavy meat-eaters to switch, while those who do opt for alternatives generally prefer plant-based options. They also noted that there remains a generally low level of knowledge about the technology, and that supplying information to potential consumers can yield mixed results, depending on the framing of that information and the prior cognitive predispositions of the respondents. This finding about the persuasive role of framing aligns with previous research on the topic in the cellular agriculture arena (29).

Interestingly, to date, significantly less scholarly attention has been paid to AFD products made through the precision fermentation process. This is despite the fact that, unlike cell-cultured meats, these products are already available for purchase in the United States, and have the potential for a swifter technological and regulatory advancement in other global contexts. A few studies have focused on the social and economic implications of AFD. Newman et al. (30) explored the potential influence of AFD on future land use change, with a particular focus on the environmental implications of using sugar as a feedstock for cellular dairy production processes. They noted that, depending on the industry's development and agricultural approach, a mix of potentially environmentally harmful or positive land use approaches could take shape.

Koch et al. (31) offered a wide-ranging scenario analysis of the global dairy industry, charting four potential futures for dairy over the course of the next decade. One of several key questions in their analysis was the potential role of precision fermentation-based dairy alternatives – across their various scenarios, they projected a range of possibilities, stretching from the technology remaining small-scale and niche up to reaching cost-effective mass market scalability. Mendly-Zambo et al. (32) provided a general overview of what they called “Dairy 3.0,” situating fermentation-derived dairy products within a broader conversation related to dairy alternatives and cellular agriculture. They highlighted questions around land use, regulation, and consumer acceptance as key areas of inquiry for scholars and practitioners interested in the topic.

In the consumer perception literature, specifically, a 2018 survey in the United Kingdom by *The Grocer*, conducted in collaboration with Harris Interactive, explored basic consumer reactions to the topic of AFD. That research found that a strong majority of respondents were unaware of the technology, and that younger consumers tended to have more positive perceptions of the concept. Taken as a whole, 28% of the sample expressed willingness to purchase what the survey termed “synthetic milk,” compared to approximately 40% who expressed outright rejection. The primary objections from respondents included concerns about potentially unsafe chemicals, unnaturalness, and possible long-term side effects (33).

In the peer-reviewed literature, Zollman Thomas and Bryant (34) conducted a large survey of respondents from across Brazil, Germany, India, the UK and the US. Their study found low levels of outright rejection, ranging from 2.1% in Brazil up to 17.2% in the US. In terms of willingness to try AFD cheese, an average of 78.8% of consumers across the five different countries indicated they would probably or definitely do so, ranging from over 90% in Brazil to approximately 65% in the US. Intentions to regularly buy the product ranged from a high of 73.9% in India to a low of 34.6% in the UK. Across all countries, higher perceptions of tastiness predicted purchasing intent, while considerations of ethics and environmental friendliness were also predictive in some, but not all, of the national contexts. Of all dietary practices with which respondents identified, flexitarianism was the strongest predictor of willingness to buy AFD products. Those with high levels of current cheese consumption tended to show the highest level of interest in trying the novel products. Compared to surveys on consumer willingness to try or purchase cell-cultured meat, openness to consuming AFD was generally more enthusiastic, which could be the result of any number of methodological, technological, or value-oriented considerations.

### Overview

Significant questions remain about the potential future of AFD, including key questions about consumer understanding and interest. With that in mind, this study set out to use focus groups to explore basic consumer perceptions on the topic. Our initial research question sought to understand how respondents make sense of a basic technological description of the AFD process, interrogating the general valence of their reaction and the key questions that would arise in response. From there, our second research question aimed to explore how respondents would react to a mix of positive and negative arguments about AFD and its implications, providing a qualitative assessment of the relative strength and weakness of these frames. The next section of this article offers more detail on the conceptual and practical elements of the methodological approach we employed, before turning to an in-depth discussion of the findings and implications.

## Materials and methods

In order to investigate these questions, we conducted 10 focus group discussions in October of 2021, with two sessions composed of respondents in the United States, Germany, and Singapore, respectively, and four sessions composed of respondents in the United Kingdom. There were several factors involved in the selection of these nations as targets, most notably a desire to have international representation while maintaining English as the primary language for data collection. In addition, consultation with industry informants identified each nation as important potential markets for AFD, albeit with varied pathways to market access. Specifically, AFD is already approved for retail sale in the United States and Singapore, while not available in Germany or the United Kingdom. All of the focus groups were held virtually via video conferencing software, due to the ongoing Covid-19 pandemic and the desire to collect data from international samples. We followed a set of best practices for virtual focus groups, including the recruitment of smaller groups of participants than is customary for in-person research, the use of a familiar video-conferencing platform (Zoom), and the employment of slides as visual aids, among other tactics (35, 36). Participant recruitment was aided by a professional global research platform (Testing Time), and participants received a monetary honorarium equivalent to €40 via that platform after their focus group concluded.

Participants were pre-screened using questions that explored their levels of interest or aversion to trying new foods, as well as their general attitudes toward the application of new technologies. In total, 42 participants took part in the focus groups, with group size ranging from a minimum of three participants to a maximum of five participants. Eight of the groups, consisting of 34 participants, focused on potential “early adopters,” a determination based on their generally favorable attitudes toward trying new foods and having a positive outlook on the role of technology in society, as measured via the pre-screening survey. This current study reports on the findings from those focus groups, excluding insights from the two “late adopter” focus groups (both of which were conducted with participants in the United Kingdom). Previous research has identified ~25–30% of society in highly developed nations can be classified as early adopters of new technologies (37). Our focus on early adopters in this current study builds upon the argument of House (38), who suggested that researchers should grant greater analytical attention to early adopters and potential early adopters when focusing on consumer perceptions of novel foods, as opposed to focusing on the general population as a whole. We agree with that author's assessment that, before industry actors or researchers can begin to think about factors that might contribute to the increased consumption of a novel food product among the general public, some degree of established consumption must first be achieved, such that it is, “the early adopters who ultimately determine if a novel food will stand or fall” (39). We determined that perspectives from late adopters, while interesting, were too limited in terms of sample size to be analyzed independently, and sufficiently dissimilar from those of early adopters such that inclusion would skew results.

An official moderator guide was developed and deployed by two different moderators across the ten total groups. An additional note-taker and assistant moderator were also in the virtual focus group to provide back-up support. All elements of the research process were approved by the lead author's university Institutional Review Board. Following the conclusion of the focus groups, the research team met to discuss whether additional early adopter sessions would be required to pursue our research questions, or whether theoretical saturation had been achieved. A review of our notes suggested new themes had not been introduced in the final set of focus groups and therefore the sample was sufficient for our study purposes.

In the initial focus group introductions, the moderator described the goal of the focus group as aiming to understand their reactions to a new type of food product, and emphasized that there were no right or wrong answers. The focus group moderator then offered a basic description of the concept and technological principles of making dairy through precision fermentation. It aimed for balanced language throughout, and solicited participants' general responses to the description, as well as asked them what key questions they had. Notably, the term “animal-free dairy” was not used in this description, nor throughout the whole of the focus group, in favor of more neutral phrases like “new type of dairy.” A final section of the focus groups, not reported on in depth in this current article, did ask participants for feedback on potential names for AFD. The full description of the food technology, displayed on-screen and read aloud by the moderator, was as follows:

*A number of companies and researchers are working to create dairy products without any animals involved. These products are not the same as plant-based milks that you might already be familiar with – like soy, almond, or oat milk. Instead, they have the same basic ingredients as milk made from animals, but the ingredients are made in a different way. In these new products, similar to beer or soy sauce production, microorganisms are used to produce the ingredients, which in the case of dairy, are the proteins whey and casein. To begin this process, a database of cow DNA is referenced, with the DNA that makes milk proteins copied and inserted into the microorganisms' genes. Through fermentation, the microorganisms start to produce proteins that are the same as those a cow would make. These proteins are collected and turned into products such as cheese, ice cream and yogurt. This new way of making dairy doesn't involve any animals, doesn't contain lactose, and tastes and behaves exactly the same as dairy we know today. Initial assessments anticipate this new way of making dairy will have a significantly reduced impact on the environment, although some think this technology may not live up to its promises*.

Following this aspect of the conversation, the focus group presented a series of visual “moodboards” that used a mix of images and brief text as a way to frame key arguments both in favor of and in opposition to the technology and its products (available as Supplementary material). This methodological strategy was guided by an understanding of two related communicative concepts: sense-making and framing. Sense-making refers to the processes by which people attempt to understand ambiguous issues and events. This meaning creation is based on current and prior interpretations of thoughts, generated by a mix of external stimuli, focused memory retrieval, and associative working memory (40, 41). The goal in sense-making research is to explore the intersecting frameworks, schemas, representations, and mental maps upon which sense-making is constructed (42).

Fundamentally, public sense-making about science and technology topics, including novel food products, occurs not through unfiltered reception, but rather through a variety of frames constructed by journalists, advocates, and other public communication professionals. Framing theory is an umbrella concept that explains how issues can be viewed from a variety of perspectives and have implications for different value sets (4). As part of a “frame contest,” one interpretive frame might gain influence over others in the mind of an individual or of broader collectives (3). Previous research has demonstrated the importance of framing for how people come to make sense of the benefits, risks, and overall value of novel foods, generally, and the products of cellular agriculture, specifically (28, 29). Through a review of scholarly research and media coverage, as well as conversations with industry and advocacy experts, the research team developed a set of positive and negative frames in order to explore participants' responses. Positive frames were presented first, with moodboards that focused on the potential value of AFD in terms of animal welfare, climate change and the environment, the overall power of technology, individual health benefits, and the reduction of public health risks, respectively. For each frame, respondents were asked the extent to which they believed these were strong or weak arguments in favor of AFD. From there, the group was asked to come to a consensus ranking, from the most convincing argument in favor to the least convincing argument in favor. This process was then repeated for the negative frames, with moodboards that focused on the potential negative potential of AFD in terms of messing with nature, creating health risks, hurting farmers, and increasing corporate power.

Following the conclusion of the focus groups, the video-recorded sessions were transcribed verbatim. Several members of the research team produced a topline report based on an initial review of those transcripts. Separately, the lead author of this article reviewed all transcripts in full and analyzed them following an adapted grounded theory approach (43). This included a multi-step coding process, moving from line-by-line open coding, to a focused coding that combined initial codes, and then to a final stage of refinement and consolidation of codes into themes. The initial coding process yielded over 800 open codes, which were then collapsed into ~75 secondary codes, which were subsequently ranked by frequency and clustered into overarching themes. Throughout the process, the motivating research questions were kept in mind, as were considerations of participant's word choice, views, intensity of feeling, levels of agreement and disagreement (44). The lead author produced initial thematic memos that were checked for validity by the other members of the research team. Following additional conversation and refinement, this process led to the confirmation of several primary thematic takeaways from the focus groups, described in full and summarized in Table 1 below. The pull quotes highlighted in the results section were identified during the coding process as illustrative of key participant insights and agreed upon by the research team as demonstrative of the thematic takeaways.

**Table 1:** Thematic categories, characteristics, and key participant questions.

**Category**	**Characteristics**	**Participant questions**
Process, safety, and regulation	• Emphasizing the need for assurances that the safety of AFD processes and products has been reviewed and approved • Prioritizing concerns about potential impacts on individual bodily health above broader public health or environmental concerns	• What exactly is in AFD and how is it created? • In what ways is AFD similar or different from genetically modified (GM) foods? • Have governmental regulatory bodies, food companies, and independent scientists assessed AFD safety?
Consumer preferences and priorities	• Expressing concern for animal welfare in industrialized animal food production • Subordinating animal welfare values to taste, price, convenience, and nutrition • Seeing AFD as a new option among many conventional, organic, and plant-based dairy products and alternatives	• What makes AFD a superior product when compared to existing dairy and plant-based dairy alternatives? • Will the nutritional profile of AFD match up to conventional dairy? • Should AFD be considered dairy? Should it be considered vegan?
Animal-free dairy, food technology, and the future	• Offering ambivalent perspectives on the relationship between food and technology • Anticipating that AFD could become a niche option within a future dairy product and dairy alternative landscape	• Is there a way to balance natural food with the use of some elements of food technology? • What would happen to dairy cows if AFD became more popular?

## Results

### Process, safety, and regulation

When encountering information about AFD, including both positive and negative frames about the technology and its impacts, respondents consistently raised questions about the technical process through which the products were created, as well as its overall safety for potential consumers. From there, they outlined the types of regulatory assurances they would need in order to support AFD's advancement. These perspectives echoed existing public opinion research, which has shown that people tend to hold official governmental regulatory agencies and food companies the most responsible for ensuring food safety, even as these institutions lag behind family members, medical professionals, and a host of other sources of food information in terms of their perceived trustworthiness (22, 45). Generally speaking, very few participants within this early adopter sample expressed strong initial opposition to the concept of AFD and its development. They did, however, have several questions and concerns, with most on the fence as to whether they would be interested in ever consuming the product themselves, pending that clarifying information. Notably, the moderator did not provide direct responses to these questions, but instead encouraged a spirit of open inquiry among participants. Ultimately, the respondents called for transparent communication from all parties involved in developing, regulating, and selling AFD, and from there believed they could make a decision as to whether it was something they were interested in consuming.

Many of the participants' requests for additional information focused on the technical aspects of AFD production. The initial descriptive text offered at the start of the focus group was seen as leaving many open questions. Participants sought to have a better understanding of the specific ingredients used in the AFD process, the role played by DNA, and other basic information about how AFD was actually made. Similarly, in response to information that suggested the benefits of AFD – including in the initial description, as well positively framed moodboards focused on the importance of acting on climate change, the health benefits of animal-free diets, and the public health risks of conventional animal products – participants wanted more concrete information that showed these claims were verified and that safety assurances were backed by independent researchers:

To make me rest assured I would need to know about the process of how the actual dairy product is produced to give me peace of mind and actually make me want to consume the product. So I think, like, transparency would be very important in this case. (Singapore Group 2)

Probes about the types of safety concerns that were important to participants demonstrated that individual bodily health – as opposed to broader public health or environmental concerns – were most salient. Here, they wanted to know exactly what type of safety testing would be conducted to ensure that AFD would not have negative human health impacts. Notably, the perceived unnaturalness of AFD was seen as a cause for potential concern. At the same time, however, a number of participants pushed back against negative framings that they felt leaned toward a fearmongering tone. Specifically, moodboards headlined with “We shouldn't mess with nature” and “We shouldn't eat what we don't understand” were seen by many as overly dramatic and disconnected from the realities of the modern food system. Fundamentally, participants tended to express faith in the judgment of official regulatory structures, including government agencies that oversaw food products and retail outlets, such that they would trust a product's safety if it was allowed to be sold in stores and restaurants:

I think it is understandable that people will be a bit afraid of something new and messing with DNA and things. But generally, I think nothing's gonna make it to your supermarket that's gonna change your DNA or kill you or whatever (UK Group 1).

Indeed, one key area in which there was significant contestation and, in some instances, outright confusion, was in discussions about how AFD was related to or distinct from other forms of genetically modified (GM) food products. The general sentiment of the focus group participants toward GM foods skewed slightly negative, and when pressed, most of those with negative perceptions of GM foods reported a general sense that they were not particularly good for individual health or the environment. Other respondents, however, pushed back on this idea, suggesting that anti-GM sentiment was overblown and not as salient as it had been in the past, pointing out that many people often eat GM products without realizing it and do not suffer negative consequences.

With this debate in mind, participants wrestled with whether AFD should or should not be considered a GM food. The initial description noted that, in the AFD process, “a database of cow DNA is referenced, with the DNA that makes milk proteins copied and inserted into the microorganisms' genes,” and one of the negatively framed moodboards made reference to concerns about genetically modified foods. The specific question as to whether or not AFD was a GM food was kept intentionally undetermined, however, in order to gauge participants' organic responses. Previous research that explores public perceptions of GM food demonstrates that understanding about the process is widely varied and attitudes about the technology can be highly charged (37). Recent research has also shown that extreme opposition to GM food tends to be associated with lower levels of objective knowledge about science and genetics, even as strong opposition is also associated with higher levels of self-reported understanding of GM food technology (46). The conversation about AFD was layered on top of this already muddled landscape. While the role of DNA and the mention of “genes” suggested to many that it was in some way connected to GM foods, others seemed to think the processes were distinct. Notably, for a number of participants, the analogy of beer brewing via fermentation made them more comfortable with the technology and helped them understand the principles of the AFD process. At the same time, a few grappled with the question as to whether AFD production was the same as traditional fermentation. Overall, the conversations demonstrated the murky terrain of knowledge and attitudes on the topic:

For me it depends. How is the food being genetically modified to begin with? We're not talking about human DNA. We're talking about a new way to prepare food. I mean, beer is fermented. I think you mentioned that earlier. Fermented milk, it sounds like a great idea, I think, but yeah. Like anything else we need extensive testing, maybe 5 years of data points, something like that (USA Group 2).

You just use the DNA to produce the milk, right? Then it is okay. But if it is genetically modified and made into a complete different version, then I'm concerned. But if it's just the copy of DNA or something, which you make the protein, then I think it's not a big deal. But it depends (Germany Group 2).

### Consumer preferences and priorities

Prompts about the potential benefits and drawbacks of AFD consistently led to broader discussions about what participants prioritized as determinants of their food purchasing and consumption. Here, respondents outlined a host of often complicated, and sometimes outright contradictory, set of food preferences, reinforcing existing research on attitude-behavior gaps and other complex dynamics in food choice (17, 18, 20). This set the stage as to whether these early adopters saw AFD as a product they would be excited to consume, be open to it as an option, or be entirely opposed to trying. While a few expressed enthusiastic support or steadfast disinterest in the product, the majority voice of the participants saw AFD as another viable choice to add to the market of dairy and its alternatives. Whether it became part of their actual eating habits would depend mostly on classic food choice factors, notably its organoleptic qualities and its price parity with standard options, a common refrain in research on alternative proteins (47).

A clear finding from the focus group discussions was that respondents were amenable to critiques about the problems of industrial farm animal production. When presented with moodboards that framed conventional practices as bad for animal welfare and harmful to the planet, many expressed familiarity with these issues, and nearly all expressed that they hoped that their eating practices would be positive on these fronts. As a response to these critiques, though, a number of participants were quick to point out distinctions between different types of animal food production, noting that animals produced in “factory farms” were too often treated poorly. A number of participants noted that they did not believe dairy production was as harmful as meat production, thus making the value proposition of AFD slightly less clear than meat alternatives. A common belief was that consumers had the power to opt for a diverse set of alternative products – indicated by official and unofficial labels such as humane, traditional, natural, local, and/or organic – that had more positive animal welfare ratings and were better from an environmental perspective:

I'm more the type that I'm trying to treat the animal better. You know, to get like a better product, not necessarily trying to find an alternative to what we have been eating (USA Group 1).

Even as they outlined concerns about animal welfare and planetary health, participants also admitted that these value-oriented propositions were often subordinated to self-oriented considerations and long-standing habits in their food choice. Here, organoleptic qualities like taste and texture, alongside price and the impact on individual health, were identified as their primary determining factors. In order to become a regular consumer, any AFD product would likely have to be better, or at the very least match, the taste and utility of conventional dairy products, as well as existing plant-based dairy alternatives. Indeed, many participants outlined that they were already consumers of alternative dairy products, either exclusively or occasionally, while others noted that they simply preferred conventional animal products and were unlikely to ever shift away from consuming them in favor of an alternative, AFD or otherwise:

We already have products to avoid eating animals, for example, there is soy and so on. So my question would be, what is the, what is the new thing about this product? If it really tastes one hundred percent like the normal dairy, then maybe I will consume it (Germany Group 1).

Participants also worked to situate AFD within broader discourses about healthy food, processed foods, and vegan animal product alternatives. A moodboard headlined “An animal free diet has health benefits” sparked spirited discussions about the nature of healthy eating, its importance to their lives, and their admitted shortcomings in terms of their own healthy eating practices. When it came to identifying and describing unhealthy food, participants consistently identified “highly processed” foods as the problem, reflecting increased public interest in “clean label” dietary trends across the globe (48). Vegan foods, many noted, could range in terms of their level of processing – several participants recounted stories of “unhealthy vegans” who lacked proper nutrition and relied too much on a diet of heavily processed foods, while others told stories of vegetarians and vegans they knew who had thrived on a healthy plant-based diet.

From there, participants also noted that much of their own diet consisted of processed foods, at least in part. While they understood that reducing processed foods would be good for them, ultimately their food choice was determined by taste, price, and overall convenience. In a number of instances, participants reflected on the own inconsistency between their ideal dietary practices and the realities of their everyday eating; many seemed to struggle with this mismatch, while others were content with admitting that they were not particularly conscious eaters:

I eat trash all the time. Like, for example, chicken that is not free range for example, we don't know what's going on, and things like processed food…So, if let's say milk is something that I don't understand, but then potentially beneficial. Why not, you know? (Singapore Group 1).

In terms of the potential health benefits of AFD, specifically, a number of participants were attracted by the lack of lactose in the product, which many noted would be useful for either themselves or close family or friends with lactose intolerance. However, they wondered whether the general nutritional profile of AFD would stack up to conventional dairy, and hoped for more information on this topic. They also wondered exactly how to categorize AFD, asking whether it should be considered vegan or plant-based at all, as well as whether it should be considered real dairy.

In sum, among this sample of early adopters, the general consensus was that participants were open to the idea of AFD as a choice. Some expressed enthusiasm about trying AFD, while many expressed a desire to taste-test it before making any further determination as to whether they were truly interested. A number of participants suggested they were fine with its development, but were ultimately happy with their existing options, be it conventional dairy or plant-based dairy. Once again, very few expressed strong opposition to the concept as a whole.

### Animal-free dairy, food technology, and the future

Several of the presented frames in the focus group prompted participants to speak more generally about their perceptions of food technology, the future of food and agriculture, and the potential place of AFD within that context. The conversations showed that participants held ambivalent perceptions about the relationships between food and technology. These findings are consistent with previous research on consumer perceptions of the food system, which has found members of the public to be divided on the risks and benefits of new food technology (37). Consumers tend to be simultaneously distrustful of “Big Food” companies (22), while also mostly satisfied with their own diet and food options (49).

In response to the moodboard headlined “Breakthrough technology makes new things possible,” for instance, several participants reflected on the positive contributions that innovation has brought, and could continue to bring, to the food system. For these participants, innovation was seen as a way to make the food system healthier, more accessible for diverse consumers, better for the environment, and better for animals. In so doing, they brought up a number of examples of food system innovations – including earlier forms of plant breeding, or new developments in alternative meats – as well as pointed to what they saw as positive innovations in other sectors, particularly the technology and consumer electronics industries. They pushed back against the moodbard titled “We shouldn't eat what we don't understand,” which included the popular missive to not eat “anything your great-grandmother wouldn't recognize as food,” by describing the many types of tasty and healthy food products they had access to that their ancestors had not, seeing that as positive progress:

I know many of the fruits we eat today, before they were cultivated, you almost couldn't eat them because the seeds were too big. So they were bred to be edible and enjoyable. So, I think ever since humans started to cultivate food, they use technology to get more out of the plants and to make the food better. So I think we always have used technology, and to improve our food and going forward, it's still going to happen, which is a good thing (Germany Group 1).

This enthusiasm was tempered, however, by a number of participants who were skeptical of positive framings about food and technology, and found negative framings about avoiding food that comes from a “chemist's laboratory,” as one moodboard put it, to be resonant. These respondents simply asserted that a mix of food and technology was not appetizing, and that we should be striving for more natural processes in the future of our food systems. Others called for a middle way, recognizing that there would certainly be elements of technology in modern food production and processing, but that we should also look for ways to retain traditional practices and natural foods moving forward:

I do think that we can do a lot of work with, you know, new technologies to develop new food and make it more eco-friendly and sustainable. However, I still think we should not totally substitute what the human touch would do. Because, you know, the best food is the easiest and more natural, et cetera. So I think a balance between them would be great (UK Group 2).

When it came to the specific role of AFD in the future of food, the vast majority of respondents, including those who saw value in the concept, did not believe that it would be a transformative technology with major social and economic impacts, at least in the short term. Negatively framed moodboards about the impact of AFD, headlined “Farmers will go out of business” and “It will mean more corporate power,” were seen as cause for concern by a few respondents, but were met by most with major skepticism. These respondents believed that issues of farmer strife and corporate power in the food system preceded AFD, caused by a host of other social and economic factors that were entirely independent of AFD and other animal product alternatives. As an aside, several respondents did wonder, if AFD *were* to achieve significant success and market share, what would happen to the cows who would no longer be needed for milking? Fundamentally, given that most respondents believed AFD would remain a niche product for the foreseeable future, they did not believe it made sense to focus on AFD as a major cause of food system concerns, even as these issues as a whole were seen as problems that needed to be addressed:

There's going to be plenty of people that will continue to eat their dairy because they love dairy. And you know, I live in Idaho where people are, we're not technologically advanced, we're very behind the rest of the world. And I think people will continue to drink dairy…This is not going to knock it out. The dairy industry, I'm not worried about, at least my fellow dairy neighbors (USA Group 1).

Ultimately, the focus group respondents acknowledged that there were many challenges in the food system, ranging from the need to produce enough food for a growing population, to concerns about health, sustainability, and animal welfare. They generally saw consumer choice as having some potential for positive impact, and believed that AFD could emerge as yet another consumer choice with potential benefits. However, several also pointed out the shortcomings of consumer-based approaches to social and environmental change, identifying “Big Food” as the real culprit of food system problems. In order for large-scale transformation to take shape, they noted, there would be a need for broader structural, governmental, and corporate changes, above and beyond what consumer choice could determine.

### Ranking the frames

Following the presentation of the positive and negative frames, respectively, respondents were asked to come to a consensus as a group to determine which of the frames were the most convincing, either in favor of or against AFD. Our analysis of these rankings is summarized in Table 2, below. The research suggests that, among the positive frames, the argument that was deemed the most pertinent was far and away claims about animal welfare. Respondents saw this as a clear problem with conventional animal food production and saw AFD as responding directly to that concern. Arguments about climate and environmental benefits ranked second, even as a number of respondents wanted more information to verify claims in this regard. Claims about the general value of breakthrough technologies, as well as the health benefits of animal-free diets, followed from there, as both were seen as connected to the potential of AFD, but also brought out feelings of ambivalence and differences of opinion. Finally, the frame that focused on broader public health benefits was ranked as the least convincing, as respondents were dubious of the moodboard in its attempt to connect animal food production with pandemic risks, and generally did not see dairy production as a major public health problem, especially when compared to industrialized meat production.

**Table 2:** Ranking frames from most to least resonant.

**Positive moodboards**	**Negative moodboards**
1. Animals deserve to be well-treated 2. We all need to act against climate change 3. Breakthrough technology makes new things possible 4. An animal-free diet has health benefits 5. Animal products carry risks to humans	1. We shouldn't mess with nature 2. We shouldn't eat what we don't understand 3. It will mean more corporate power 4. Farmers will go out of business

In terms of the negative frames, the two moodboards that focused on messing with nature and creating potential health risks to individuals were seen as the strongest arguments against AFD. While this group of early adopters was not wholly convinced by these frames, they saw potential risks in these arenas, and also saw ways that opponents of AFD could effectively leverage these concerns when discussing the technology with the broader public. As previously noted, frames about risks to farmer economies and the further consolidation of corporate power were generally seen as unconvincing. This was not because respondents did not see these as problems at all, which some did, but rather because they just did not imagine AFD would be a strong enough force to have major impacts. Taken as a whole, following the presentation of both positive and negative frames, the general sentiment of participants toward AFD was moderately positive. With that said, a number of significant questions remained about both the process and the product, and the extent to which their perceptions would be swayed further in one direction or another would largely depend on future information and direct experiences.

## Discussion

Among our sample of potential early adopters, the general consensus was a cautious openness to the idea of AFD. Outright opposition to the concept was rare, but so too was unabashed enthusiasm. Instead, respondents had a number of questions about the nature of the technological process, its overall safety and regulatory standards, its potential contributions to individual health and climate change mitigation, as well as its organoleptic qualities and price to consumers. Through these conversations, they grappled with their own ambivalence about eating animal products, as well as their mixed feelings regarding the role of technology in food. They pushed back against what they felt were overly hyperbolic claims, both in favor of and against AFD, and called for transparent communication from all parties. Participants tended to understand that much of the food we eat today has changed over time, and saw challenges to small-scale food producers as long-standing, subject to global trends, and often due to competition with industrial food producers, not alternative protein products. Most doubted that AFD would become a dominant part of the dairy market in the years ahead, but could see it finding some consumer base, if it tasted good enough and its claimed benefits could be verified. Lingering concerns about the role and impacts of genetic modification were present as well, and while analogies to beer brewing and other traditional forms of fermentation were seen as assuring for many, some still had their reservations, and could see how any mention of “genes” might be a deal breaker for other people in their social orbit.

This research offers a useful contribution to the scholarly literature on food choice and public perceptions of novel food technologies, generally. It provides confirmation of long-standing conclusions in that field, particularly in that it demonstrates the complex and sometimes internally inconsistent ways that people think about the motivations for their eating behaviors (18, 20). Within research on animal product alternatives, it represents one of the first ever explorations of consumer perceptions of AFD, a curious gap considering its relatively advanced technological and market-based status (34). Here, it provides further confirmation of the potential power of framing effects in shaping public reactions to alternative proteins (29), as well as the vital importance of taste and price parity for the products' viability as a consumer option (47).

For companies at the forefront of introducing AFD to the market, this research highlights that beyond satisfying the sensorial, functional, and price expectations of consumers, thoughtful and inclusive discourse with society will be central in determining consumer interest in AFD. In this way, companies should understand their role not merely as contributors to already packed grocery aisles, but also as participants in a discussion about our relationship with the food we eat and the future of food system reform. Specifically, the importance of transparency and clarity was apparent throughout the process, and it is already a key issue that groups like the Non-GMO project are identifying as a central reason for opposition. Although focus group participants did see a significant role for regulators and retailers in helping them gauge the safety of novel food products like AFD, companies would be wise to proactively engage with consumers to explain the nature of precision fermentation and the principles of synthetic biology underlying it, as well as advocate for robust and trustworthy official regulatory processes.

The relationship between AFD and GM foods will continue to be a hot-button topic of conversation and contestation. From an industry perspective, there is clear value in forthright communication that outlines the scientific overlaps and distinctions between these technological approaches. Avoiding this discussion entirely could very well create more opportunities for opponents to attack AFD, effectively linking a lack of transparency to a broader culture of distrust of Big Food (22). Meanwhile, advocates who are skeptical of AFD's use of synthetic biology should be mindful of the effects of their communication as well. While global attitudes toward GM foods remain generally negative, there is recent evidence to suggest that much of the general public is relatively neutral to the process, and that the most ardent opponents often have basic misconceptions about the objective science of the topic (46). Hyperbolic claims about the dangers of AFD could resonate with a niche group of highly motivated technological skeptics, but could also cloud the overall quality of the communication environment, and a lack of credibility could potentially undermine the broader goals of those who promote lower-tech future food approaches. A shared goal should be to accurately contextualize AFD technology and give consumers the opportunity to assess varied arguments in a straightforward manner. In many respects, the focus groups aimed to provide a platform for exactly this type of discourse, and participants expressed appreciation throughout the discussions for the opportunity to think through the processes and implications of AFD. In this particular sample of early adopters, that conversation often led to moderately positive perceptions of AFD by the conclusion of the group, but that may not be the case with all participants.

For organizations engaged in advocacy to shift food system practices, a particularly notable finding centered on the power of animal welfare framings as a potential benefit of AFD. In the focus group conversations, the animal welfare frame was seen as the most compelling reason to support AFD; further, in subsequent discussions about nomenclature preferences, the name “animal-free dairy” was consistently found to be superior to others, in that it highlighted the benefits of removing animals from the dairy production process. In recent years, advocates for animal product alternatives have often shied away from focusing on farmed animal suffering in their public communication, believing that consumers would be more motivated to reduce animal product consumption or try alternatives based on health or environmental concerns. Despite these assumptions, a growing body of research demonstrates that animal cruelty-focused messages might be the most powerful for actually shifting attitudes and promoting dietary behavior changes away from conventional animal foods (50). This may not be an ideal marketing strategy for companies developing and selling AFD and other alternative animal products, but it does speak to the potential for future collaborations between industry and farmed animal welfare advocates. While many people choose alternative protein products after reflecting on the implications of animal food production, the reverse may also hold true, such that the existence of appealing alternatives to animal-derived products could prompt consumers to reflect on the problems of animal welfare in food production, and from there examine the implications of their dietary choices. With this in mind, campaigns that coordinate shared messaging and leverage the power of opinion leaders on social media platforms could emphasize the animal welfare problems of conventional practices while touting the advantages of AFD and other alternatives in this domain.

The work presented in this article is necessarily limited by elements of the focus group research approach. Notably, the small sample size and focus on early adopters could constrain overall generalizability. The fact that the group conversation was driven by the researchers' interests and conceptual framing might have encouraged biases in the data, including social desirability and forced response. Related, while peer-to-peer conversational dynamics are often seen as a strength of focus group research, it can also emerge as a limitation if it encourages group conformity; while lack of variance in perspectives did not appear to be a major issue in these focus groups, the concern remains worthy of consideration (51). In addition, the virtual environment did lead to a few technical difficulties and internet connectivity problems for both moderators and participants, and at times might have led to distracted participation. Overall, consistent with previous research, effective preparation and management allowed for these obstacles to be overcome (35). Future scholarship should use a mix of qualitative, quantitative, and experimental methods to explore consumer perceptions of AFD with diverse samples, as well as to trace broader media coverage and public discourse on the topic.

As noted throughout this paper, the key to AFD's future as a viable market option will depend in large part on the extent to which it can clearly demonstrate that it is preferable to conventional dairy or its plant-based competitors. In addition to meeting the taste preferences of consumers, the industry and its advocates should be forthright and transparent in terms of the science underlying AFD, as well as its health and safety implications, nutritional components, environmental effects, and animal welfare implications, all as a means to allow a clear public assessment. This means ongoing conversations and cooperation with governments, regulators, incumbent players (both small and large), and advocacy organizations with varied perspectives on the risks and benefits of AFD. The findings of this current research demonstrate that members of the eating public take the flaws of conventional dairy production into consideration and are open to having a conversation about AFD. The way that conversation plays out will go a long way toward determining whether and how AFD becomes a significant part of the future dairy ecosystem.

## Data availability statement

The raw data supporting the conclusions of this article will be made available by the authors, without undue reservation.

## Ethics statement

The studies involving human participants were reviewed and approved by Fordham University Institutional Review Board. The patients/participants provided their written informed consent to participate in this study.

## Author contributions

GB, OZ, and CD: conceptualization, research design, data collection, and writing manuscript. GB: ethics application and data analysis. DB: data collection. BL: research design and data collection. All authors contributed to the article and approved the submitted version.

## Funding

A grant from Formo to Mercy For Animals provided financial support for the research. Funding was used for payments to focus group participants and open access publication fees. None of the funding was used to compensate the authors of this paper directly.

## Conflict of interest

Author OZ was a researcher for Formo, an animal-free dairy company. CD was a researcher at Mercy For Animals, a farmed animal welfare organization. DB was a research fellow with Mercy For Animals. BL was a researcher for Formo. The research was overseen by GB and conducted in the absence of any other commercial or financial relationships that could be construed as a potential conflict of interest.

## Publisher's note

All claims expressed in this article are solely those of the authors and do not necessarily represent those of their affiliated organizations, or those of the publisher, the editors and the reviewers. Any product that may be evaluated in this article, or claim that may be made by its manufacturer, is not guaranteed or endorsed by the publisher.
