# Supplementary material for: Framing the futures of animal-free dairy: Using focus groups to explore early-adopter perceptions of the precision fermentation process
Source: Front Nutr. 2022 Oct 3;9:997632. doi: 10.3389/fnut.2022.997632 (PMC9574361; doi:10.3389/fnut.2022.997632)
Supplement: Supplementary file 1 [file Data_Sheet_1.PDF]

MODERATOR BOOK

1.
NEW WAY TO MAKE DAIRY PRESENTATION

A New Way to Make Dairy

A number of companies are working to create dairy products without any animals involved.

These products are not the same as plant-based milks that you might already be familiar with -- like soy, almond, or oat milk. Instead, they have the same basic ingredients as milk made from animals, but the ingredients are made in a different way. In these new products, similar to beer or soy sauce production, microorganisms are used to produce the ingredients, which in the case of dairy, are the proteins whey and casein.

To begin this process, a database of cow DNA is referenced, with the DNA that makes milk proteins copied and inserted into the microorganisms' genes. Through fermentation, the microorganisms start to produce proteins that are the same as those a cow would make. These proteins are collected and turned into products such as cheese, ice cream and yogurt.

This new way of making dairy doesn't involve any animals, doesn't contain lactose, and tastes and behaves exactly the same as dairy we know today.

Initial assessments anticipate this new way of making dairy will have a significantly reduced impact on the environment, although some think this technology may not live up to its promises."

2.
POTENTIAL POSITIVES

Animals deserve to be well-treated

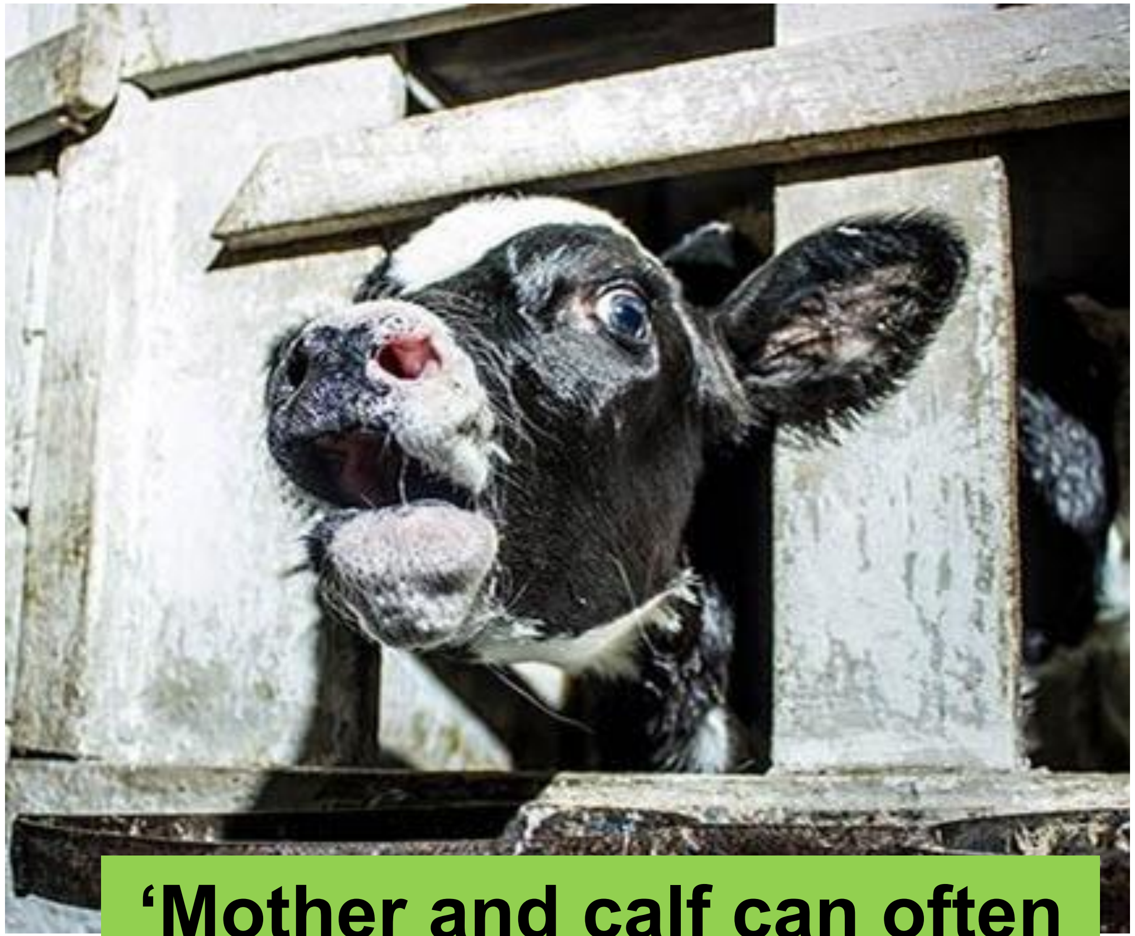

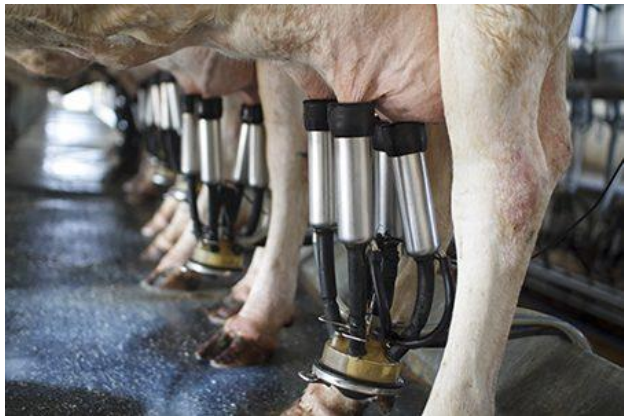

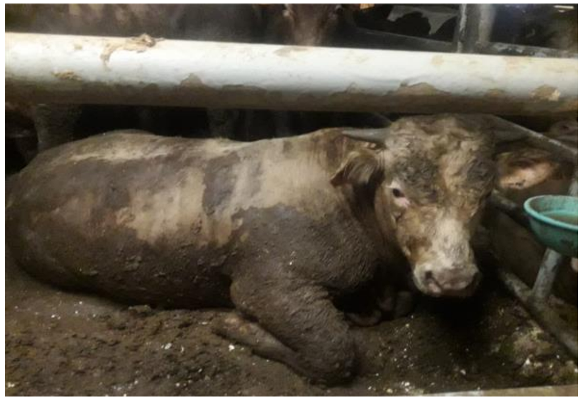

A cow can live for around 20 years, but in commercial systems she will be culled at 6 years old, on average.

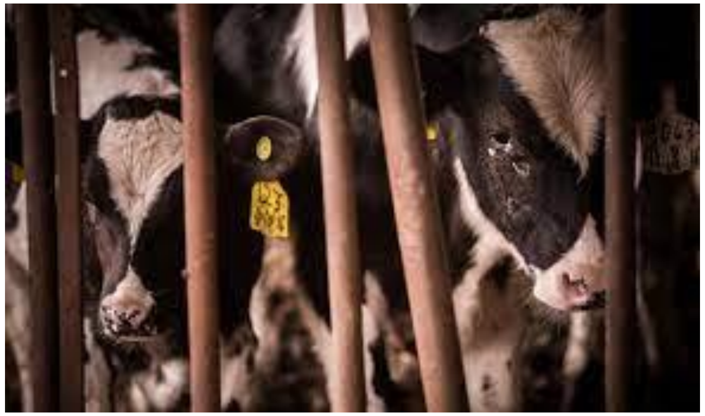

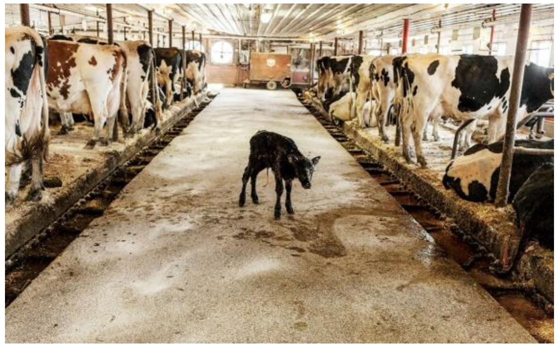

'Mother and calf can often be heard calling out to each other for hours.'

We all need to act against climate change

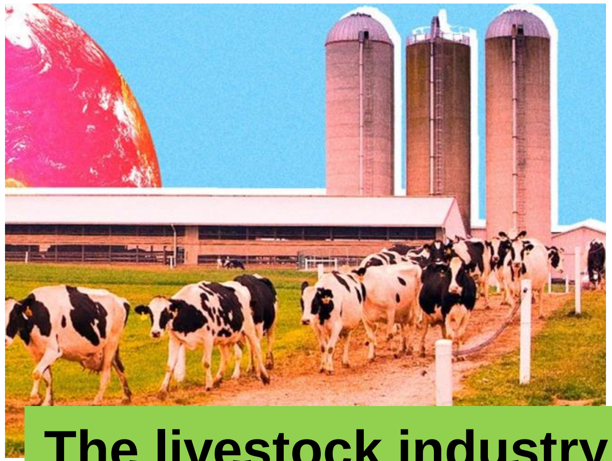

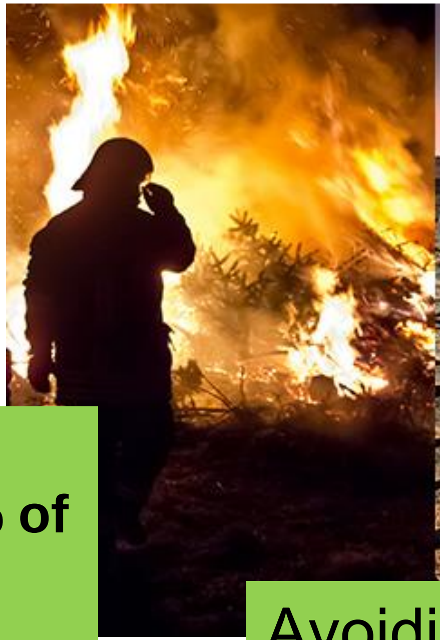

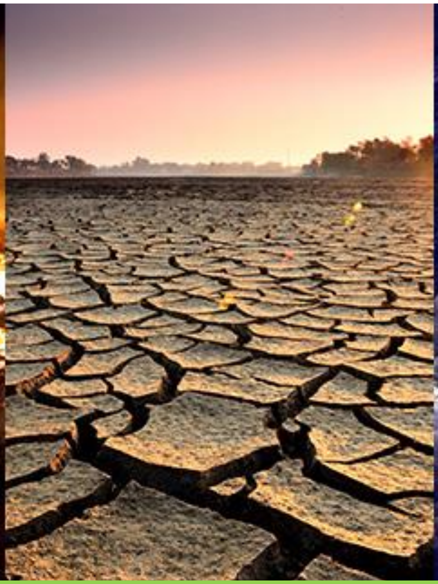

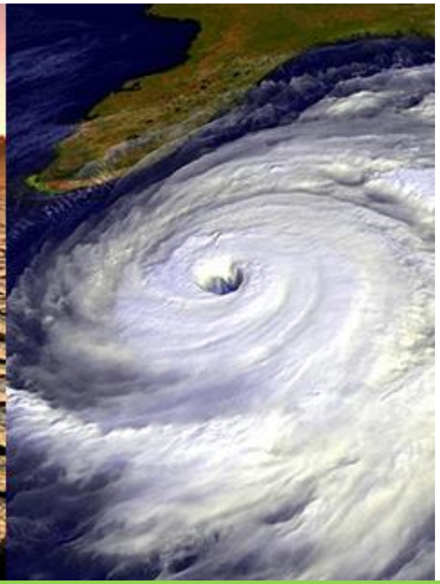

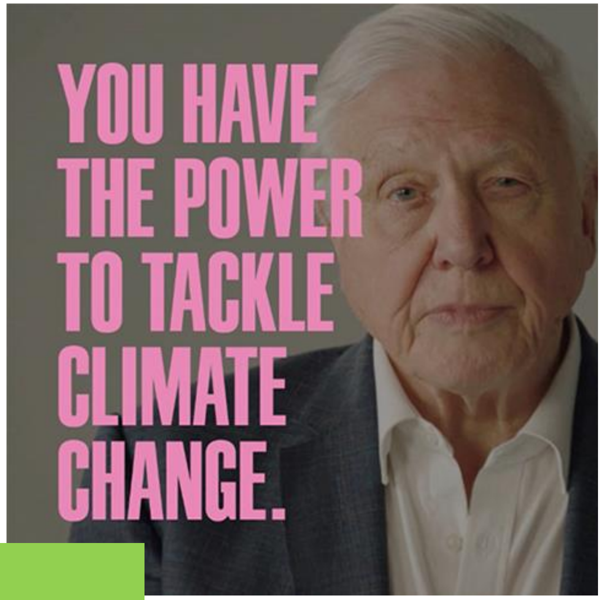

The livestock industry produces at least 14.5% of total greenhouse gas emissions.

Avoiding meat and dairy is ‘single biggest way’ to reduce your impact on Earth.

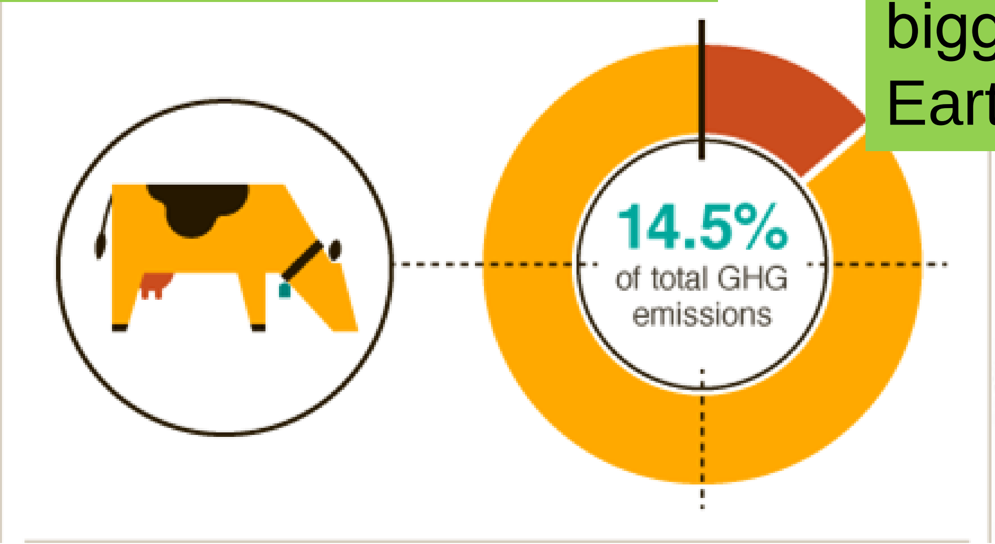

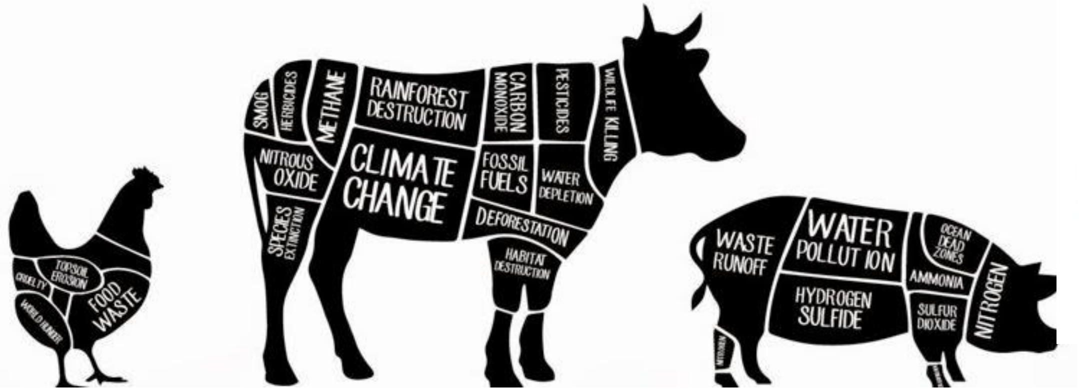

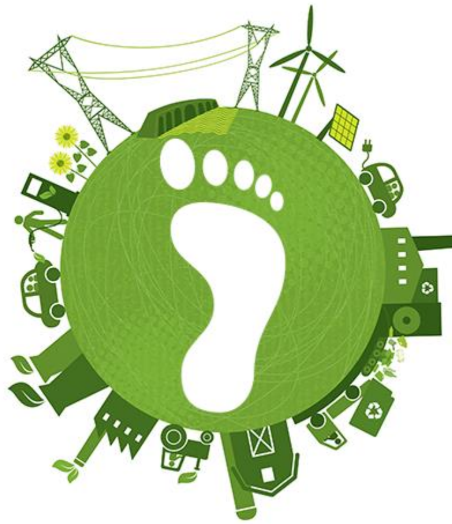

Breakthrough technology makes new things possible

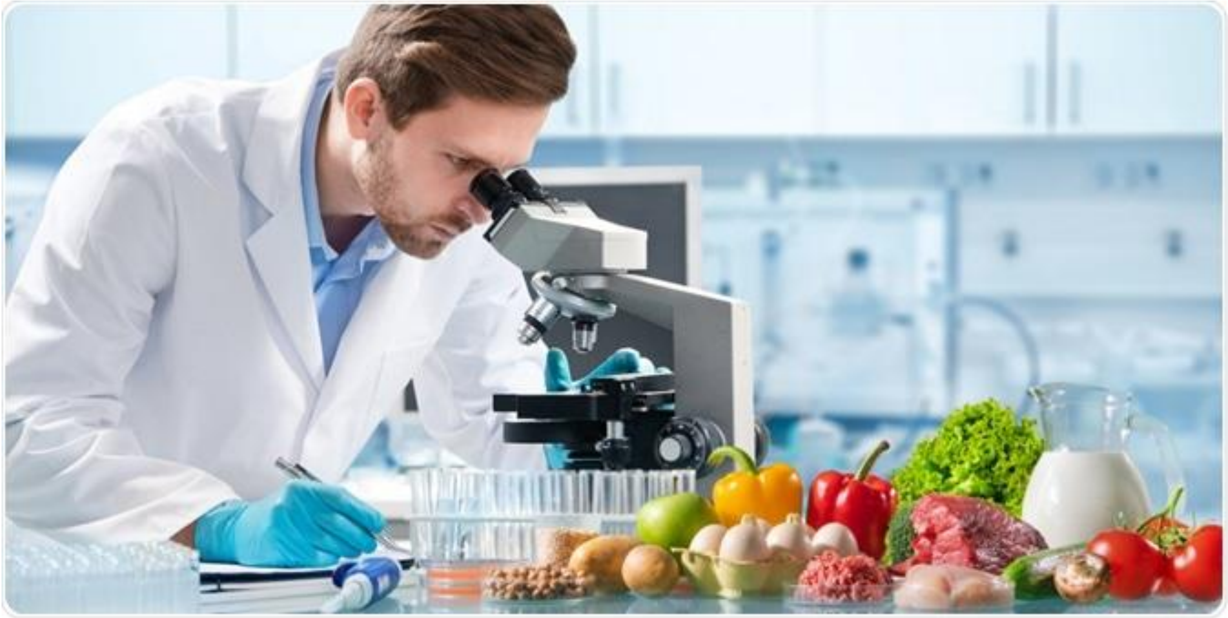

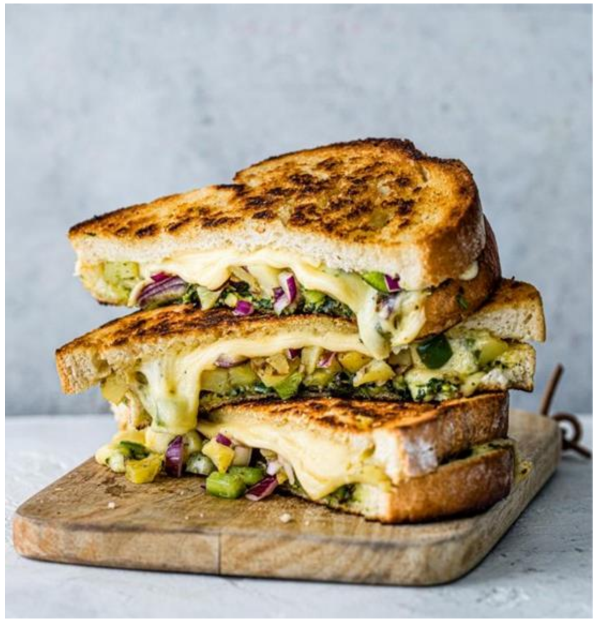

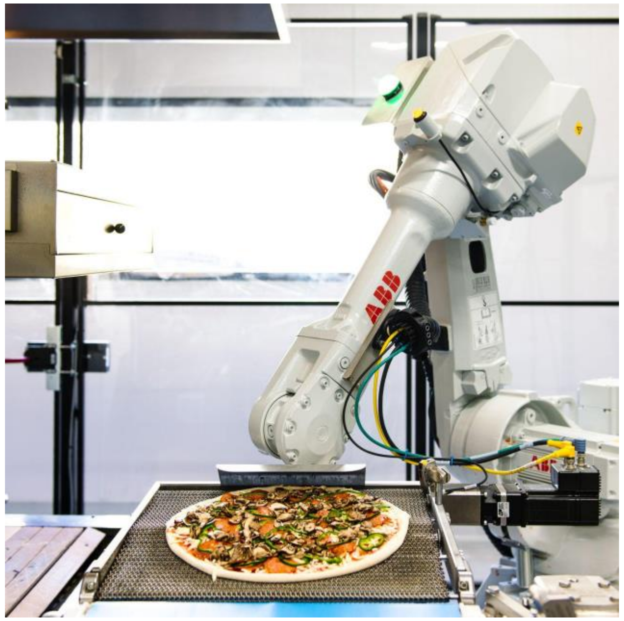

‘Advances in food technology mean we can finally align our consumption with our morals.’

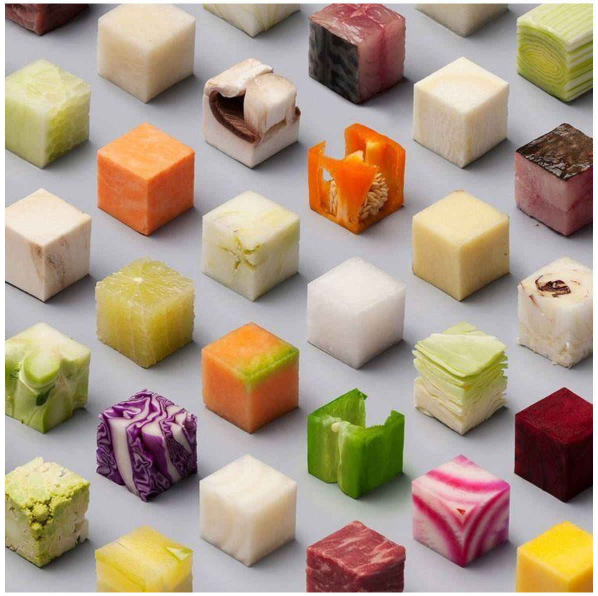

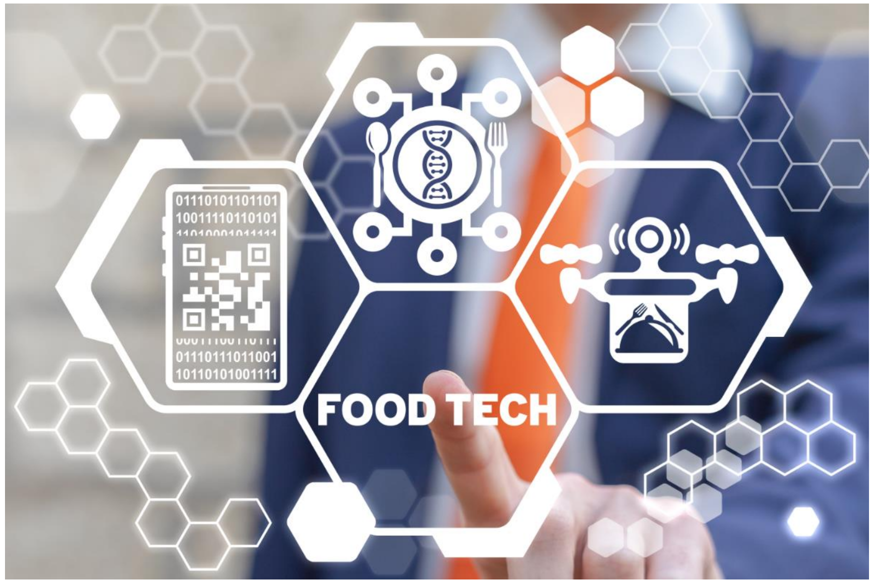

‘The technology being created now is the future of food production.’

An animal-free diet has health benefits

‘A vegan diet can help prevent heart disease and diabetes while maintaining a healthy BMI.’

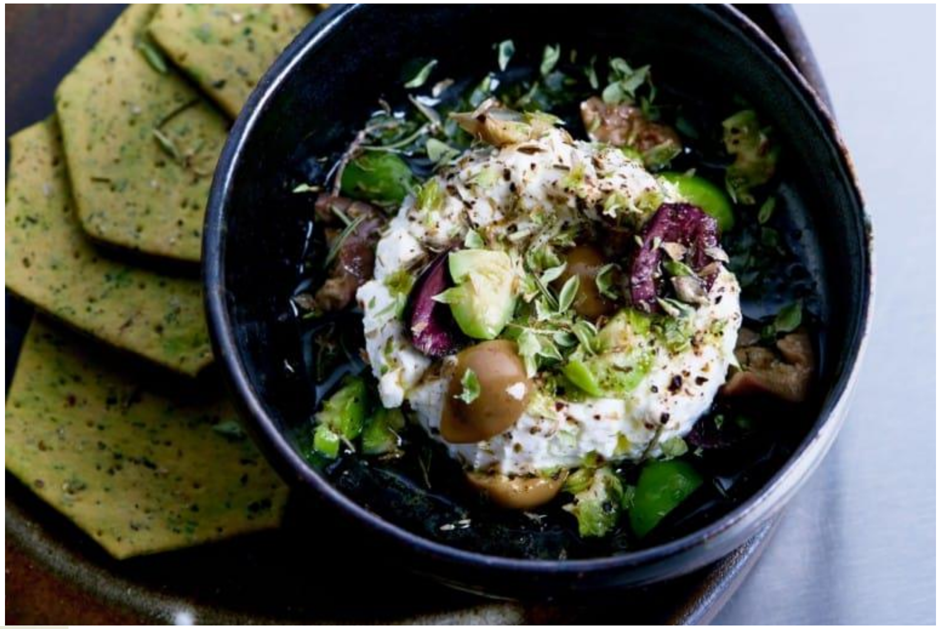

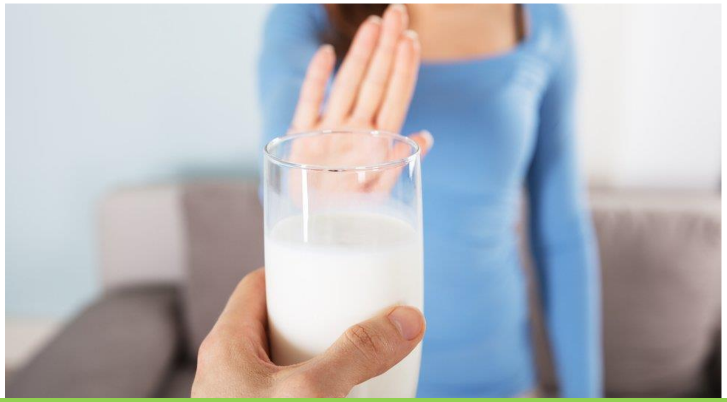

Around 60% of adult humans worldwide have reduced tolerance to lactose.

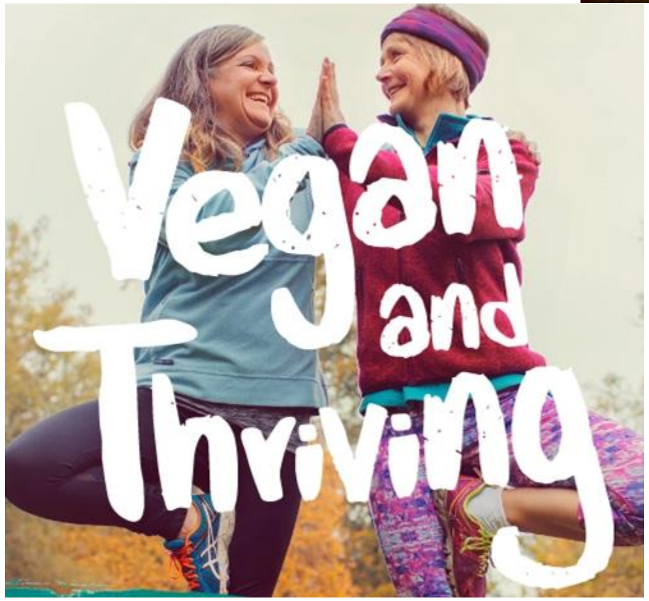

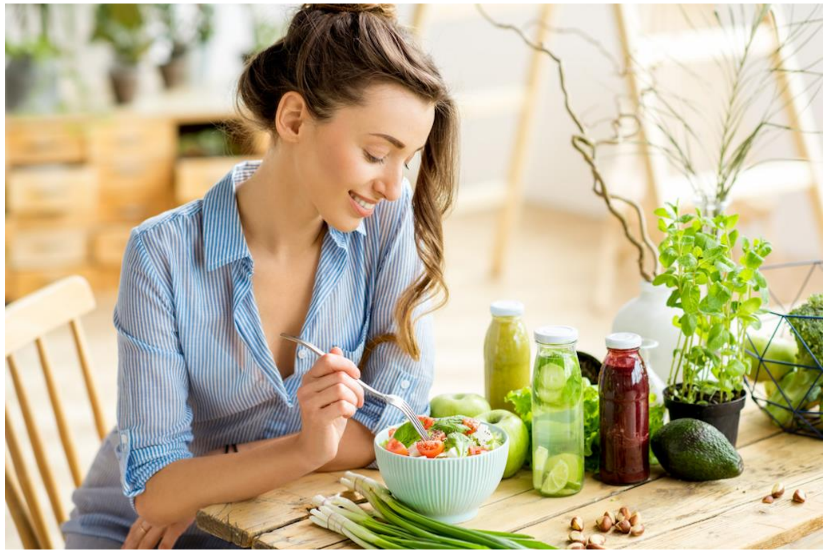

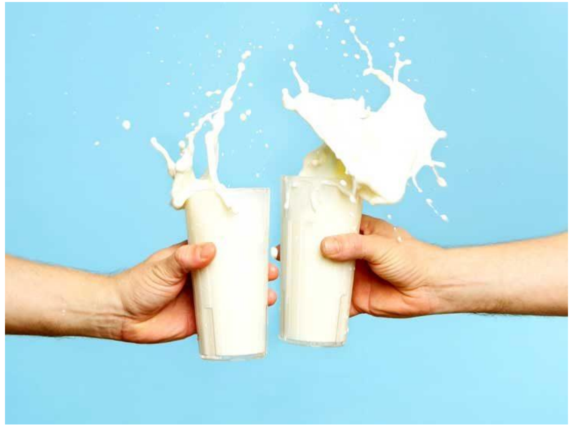

Animal products carry risks to humans

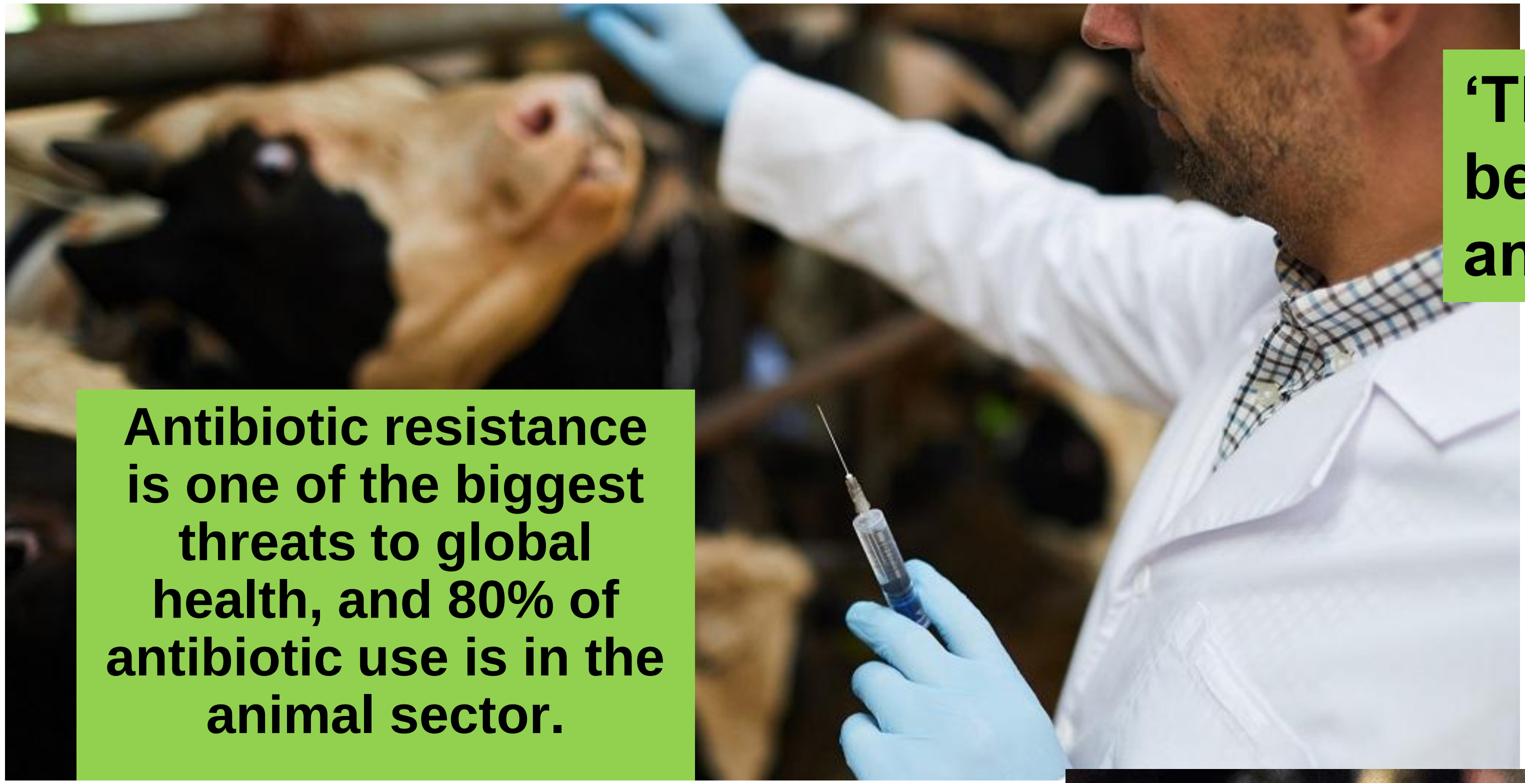

Antibiotic resistance is one of the biggest threats to global health, and 80% of antibiotic use is in the animal sector.

'There is a causal relationship between eating animal products and viral epidemics.'

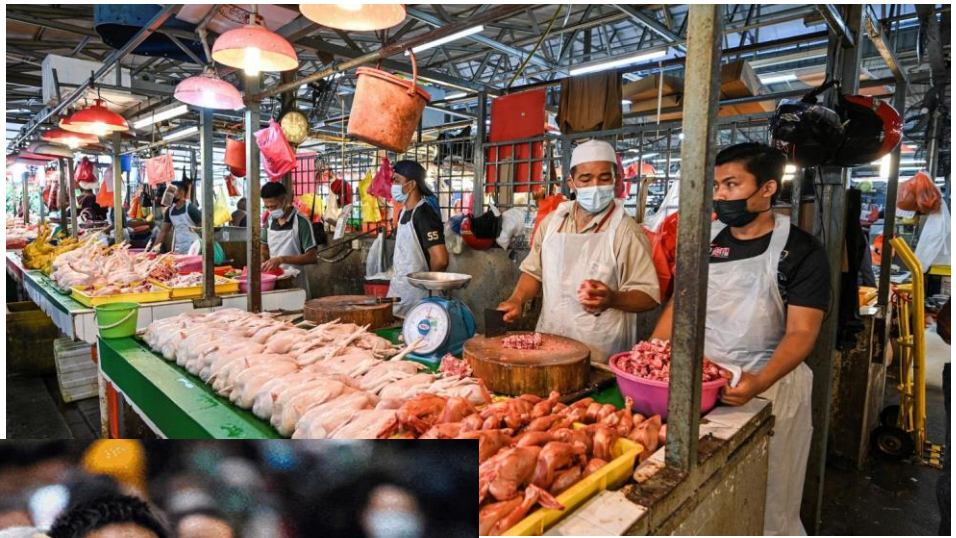

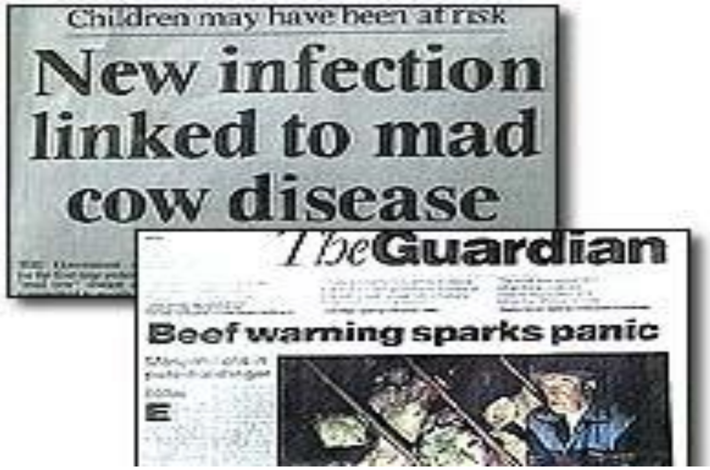

Children may have been at risk

New infection linked to mad cow disease

The Guardian

Beef warning sparks panic

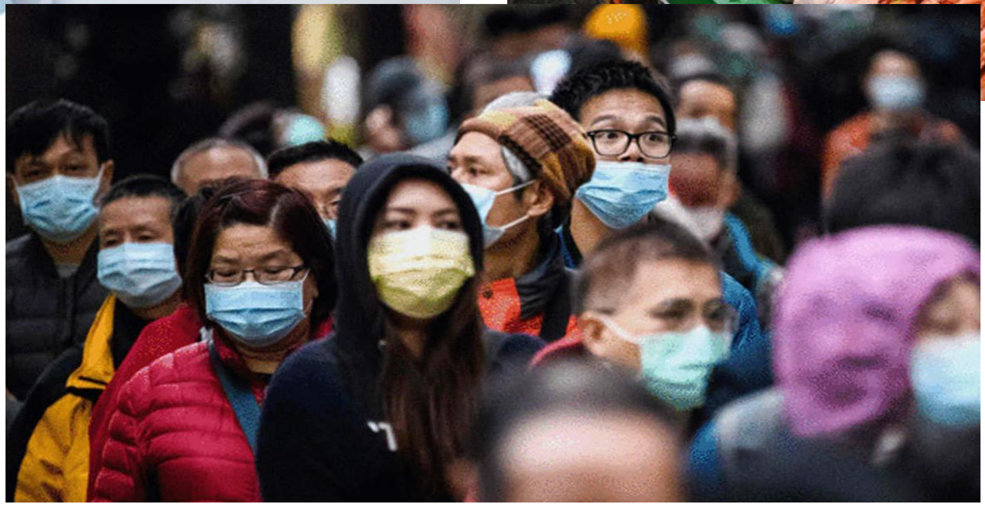

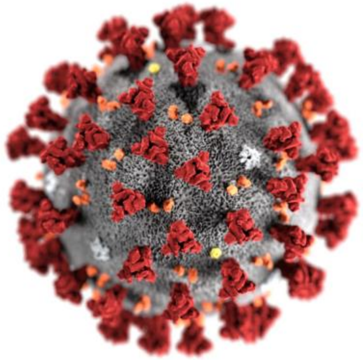

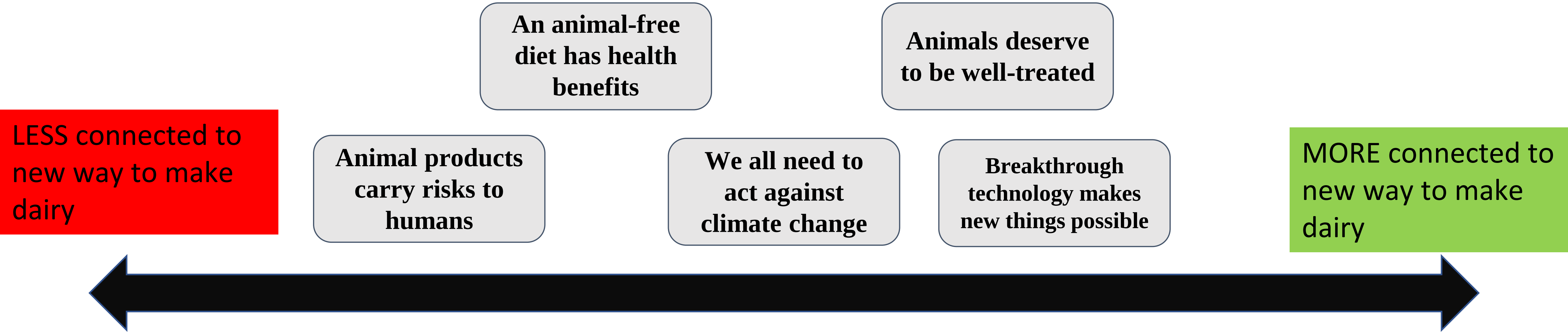
An animal-free diet has health benefits
Animals deserve to be well-treated
LESS connected to new way to make dairy
Animal products carry risks to humans
We all need to act against climate change
Breakthrough technology makes new things possible
MORE connected to new way to make dairy

3.
POTENTIAL NEGATIVES

We shouldn't mess with nature

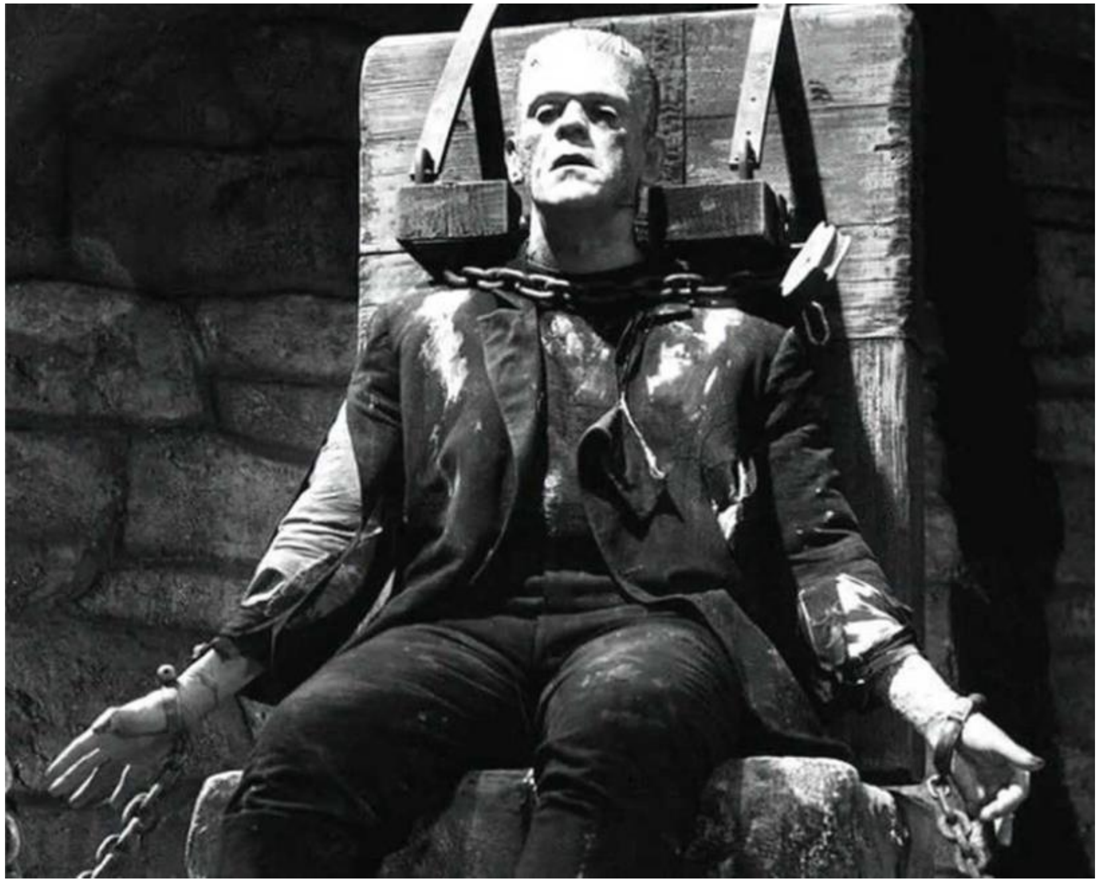

'Changing DNA is playing God!'

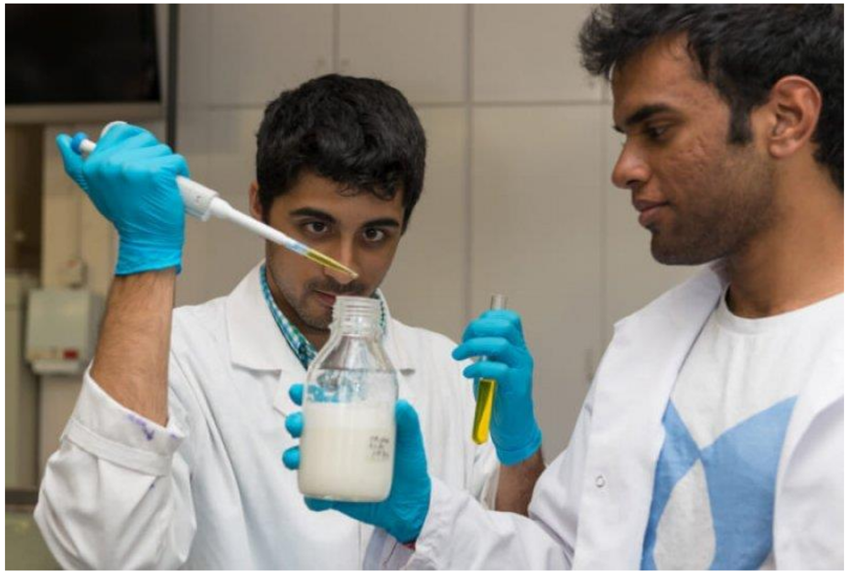

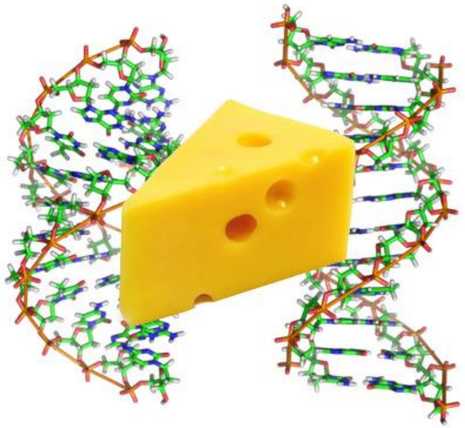

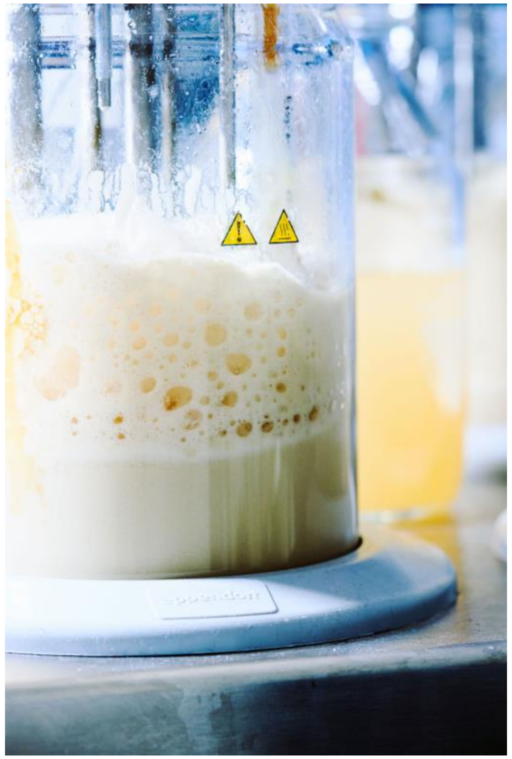

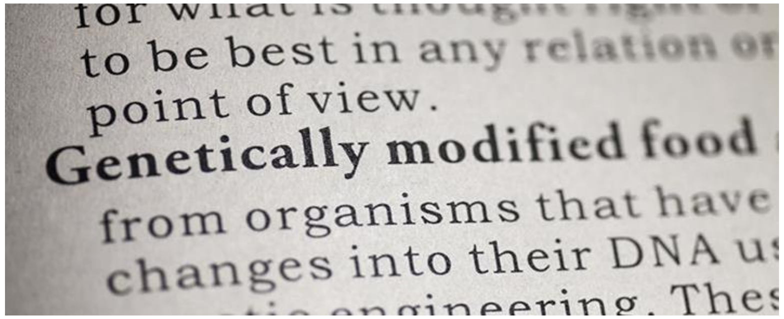

'What happens when this stuff escapes the lab?'

We shouldn’t eat what we don’t understand

‘Don’t eat anything your great-grandmother wouldn’t recognise as food.’

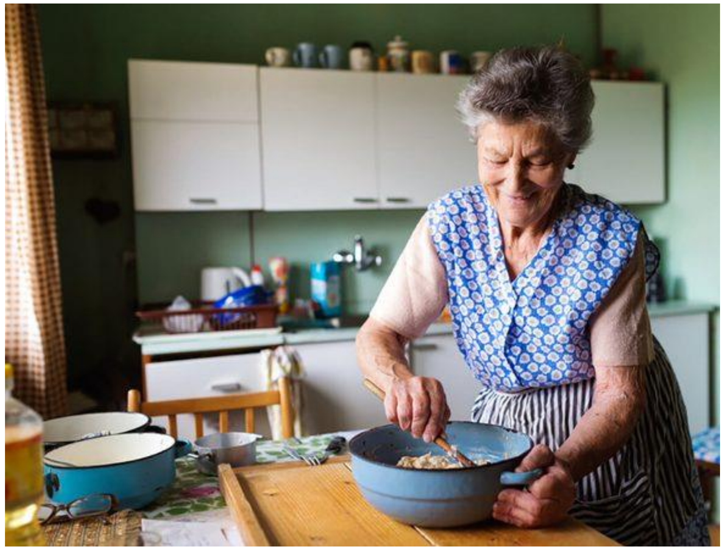

‘Eat food that comes from a farmer’s field and not a chemist’s laboratory!’

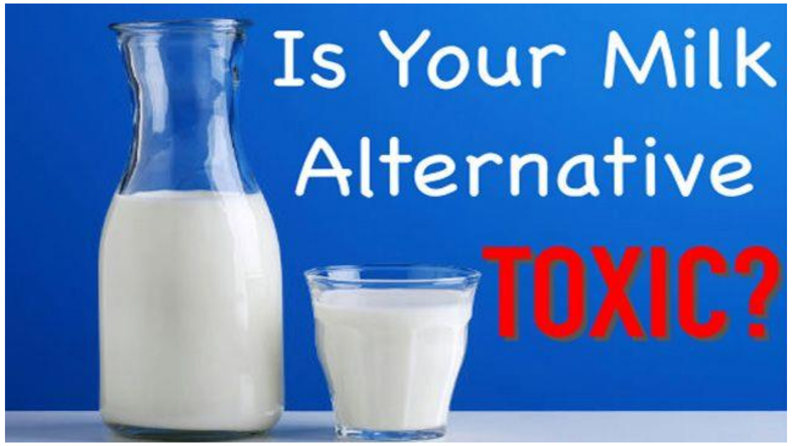

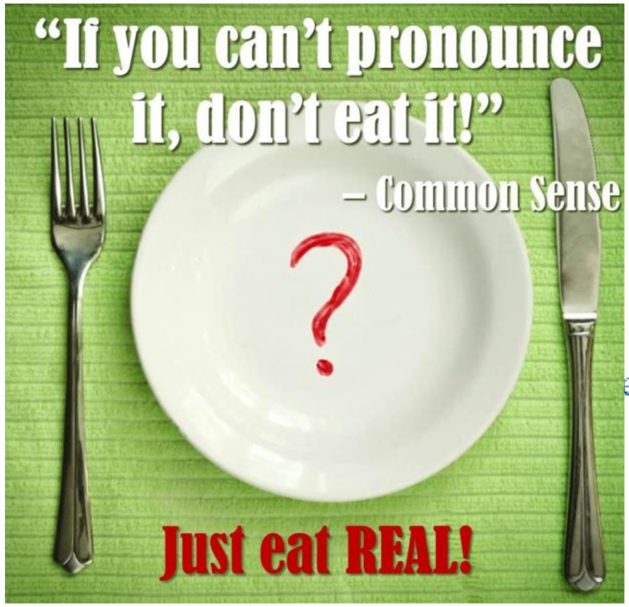

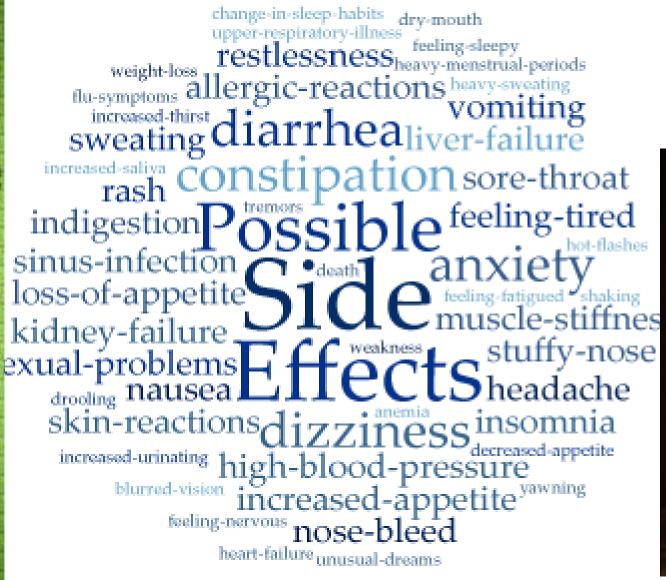

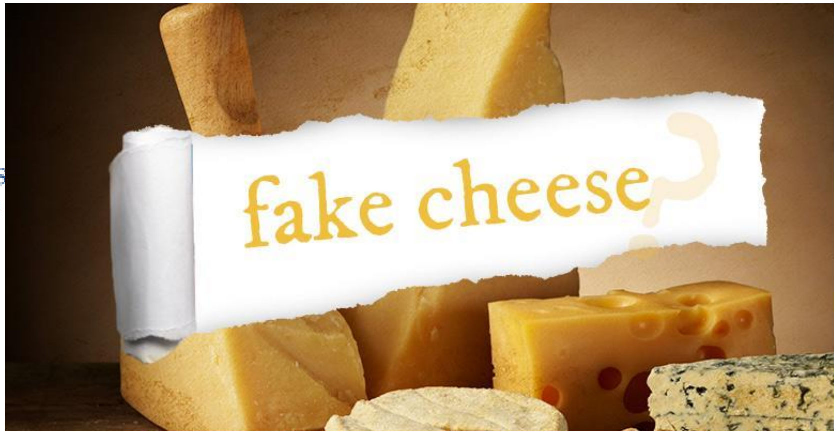

Farmers will go out of business

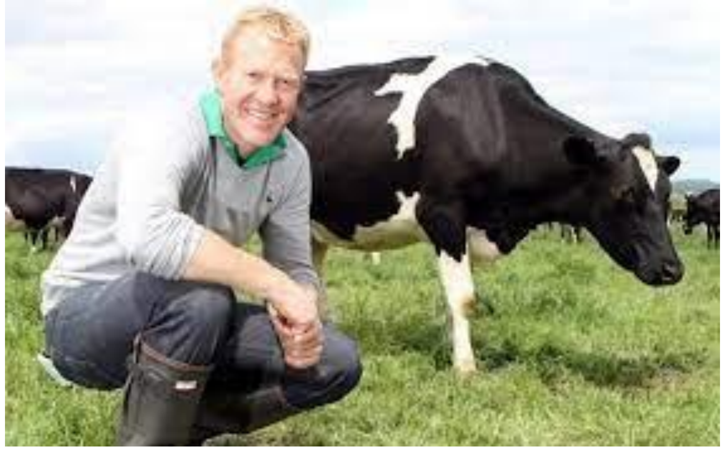

‘**Bankruptcies are up 24% from last year. Dairy farmers are struggling.**’
National Farmers Union

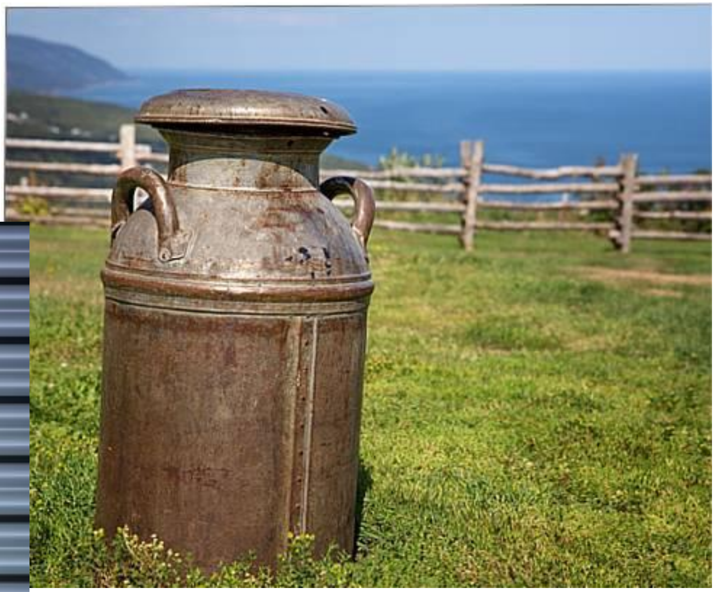

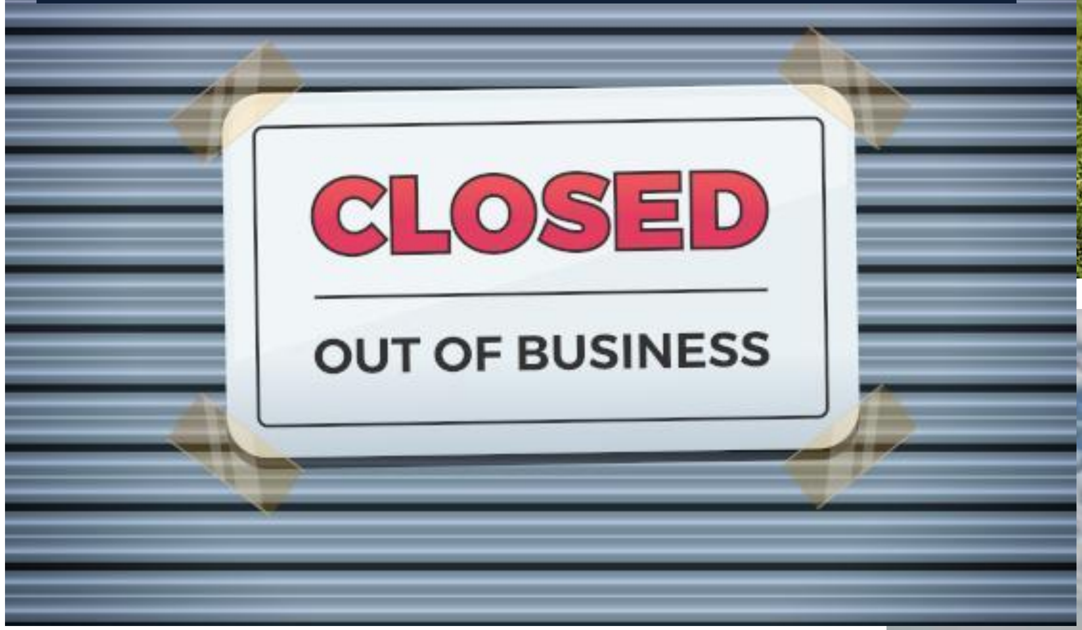

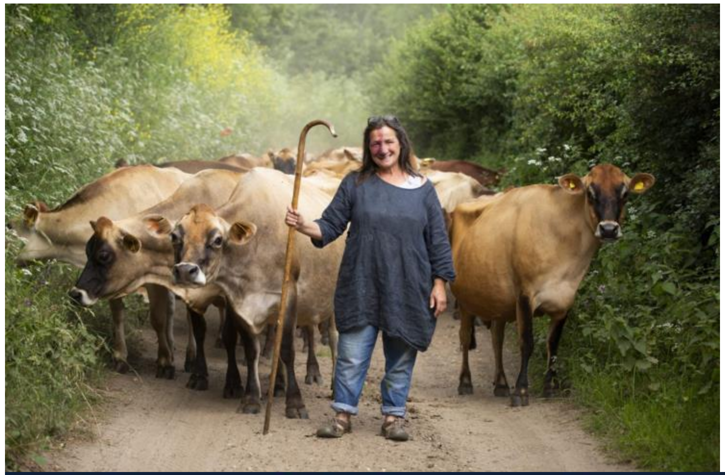

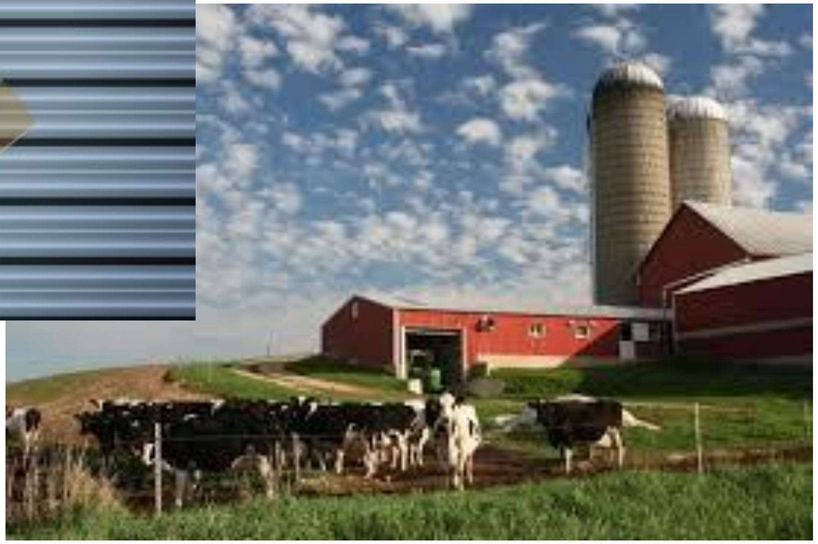

Fake milk is real news, as synthetic alternatives threaten traditional dairy farms

It will mean more corporate power

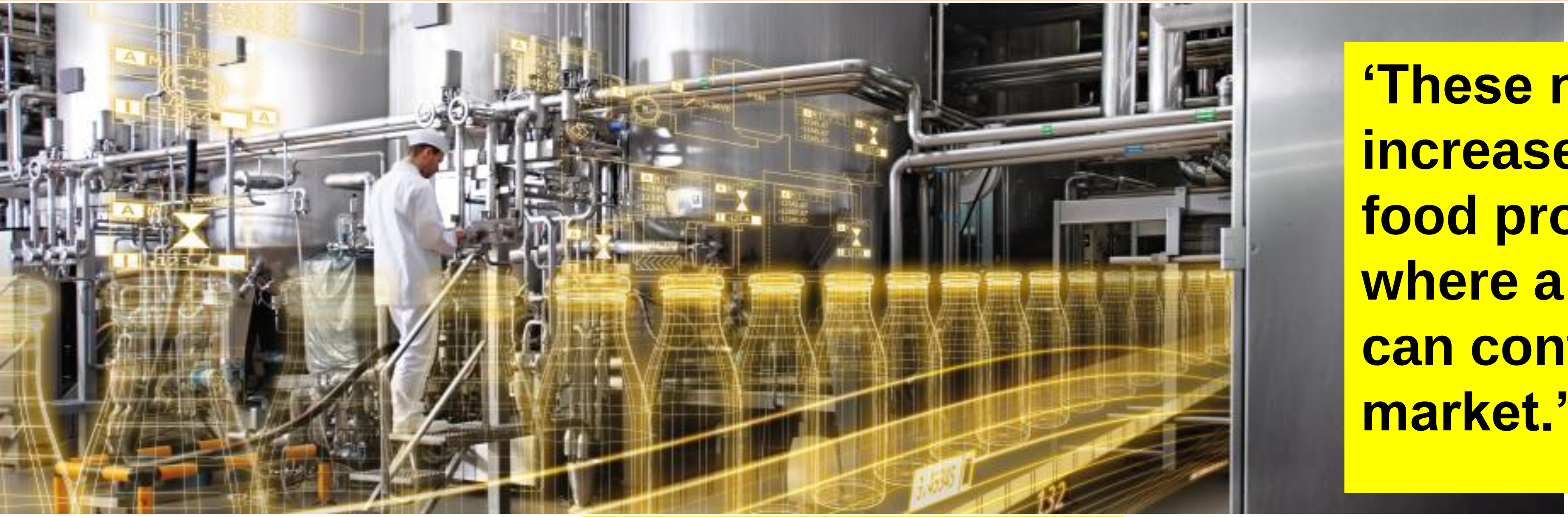

‘These new methods could increase the efficiency of food production to the point where a few big companies can control the entire market.’

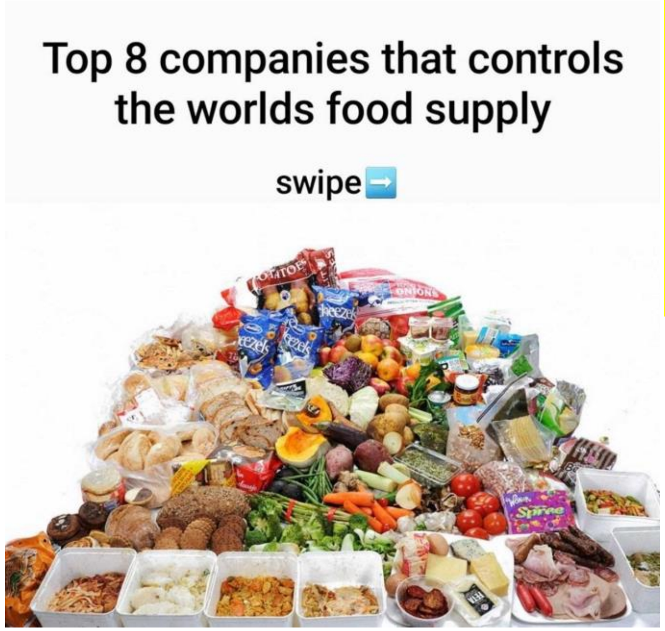

‘Who controls the food supply controls the people.’

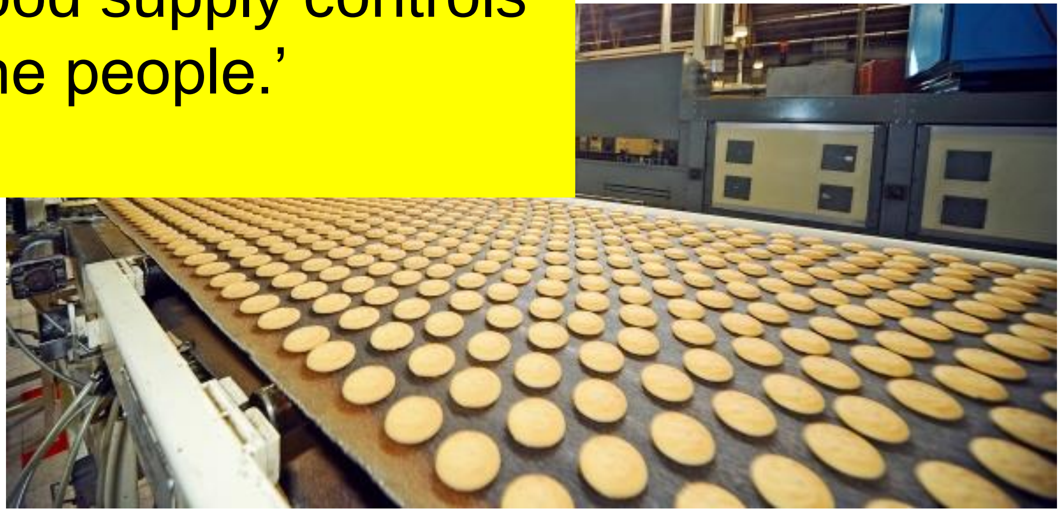

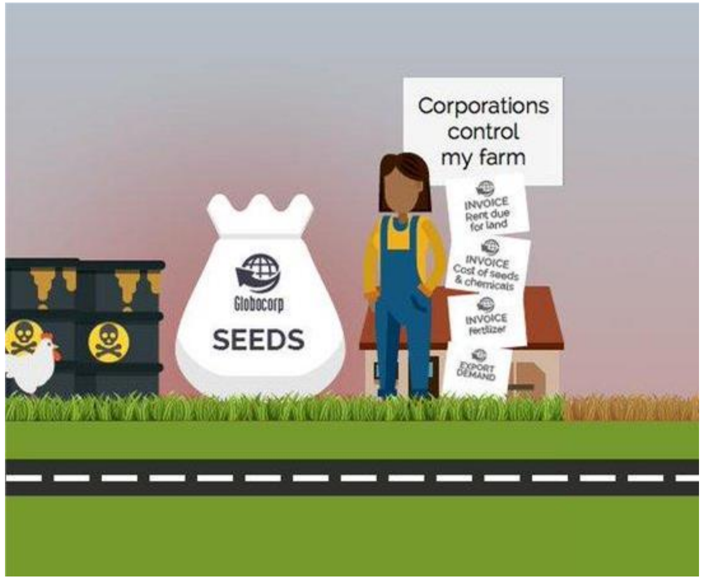

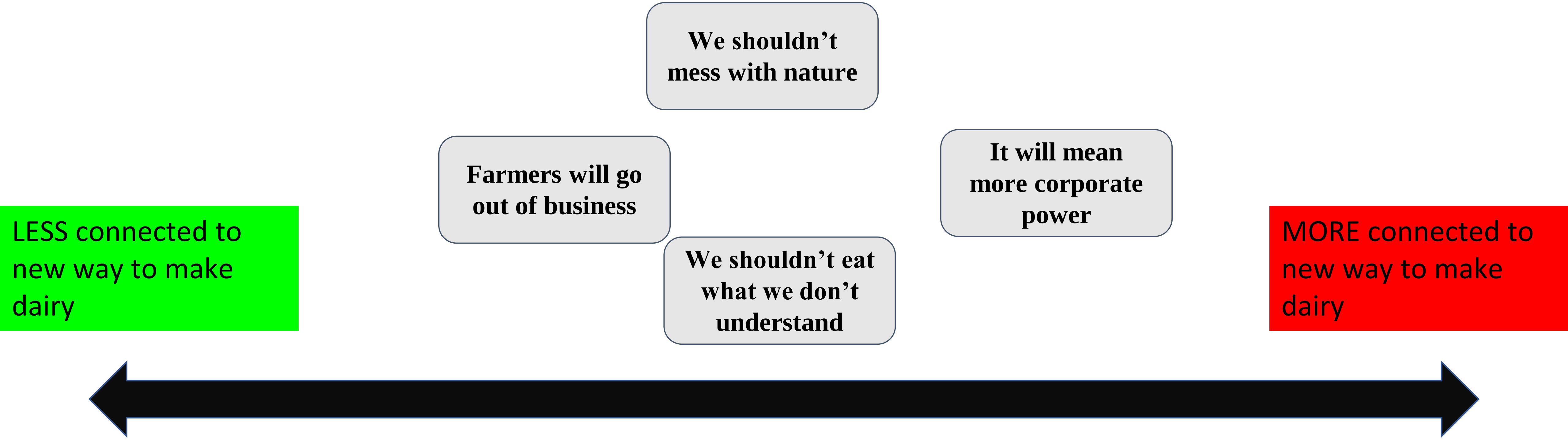
We shouldn't mess with nature
Farmers will go out of business
It will mean more corporate power
LESS connected to new way to make dairy
We shouldn't eat what we don't understand
MORE connected to new way to make dairy
